# Cryo-Induced Cellulose-Based Nanogel from *Elaeis guineensis* for Antibiotic Delivery Platform

**DOI:** 10.3390/ijms24021230

**Published:** 2023-01-08

**Authors:** Tasnim Hajidariyor, Nutchanon Nuntawad, Panadda Somsaen, Raninnart Prukdamrongchai, Harit Cherdchoo, Pattaraporn Posoknistakul, Pongtanawat Khemthong, Wanwitoo Wanmolee, Pariyapat Arjfuk, Pisut Pongchaikul, Navadol Laosiripojana, Kevin C.-W. Wu, Chularat Sakdaronnarong

**Affiliations:** 1 Department of Chemical Engineering, Faculty of Engineering, Mahidol University, Nakorn Pathom 73170, Thailand; 2 National Nanotechnology Center (NANOTEC), National Science and Technology Development Agency (NSTDA), Pathum Thani 12120, Thailand; 3 Chakri Naruebodindra Medical Institute, Faculty of Medicine Ramathibodi Hospital, Mahidol University, Samut Prakarn 10540, Thailand; 4 The Joint Graduate School of Energy and Environment, King Mongkut’s University of Technology Thonburi, Bangkok 10140, Thailand; 5 Department of Chemical Engineering, National Taiwan University, Taipei 10617, Taiwan; 6 Center of Atomic Initiative for New Materials (AI-MAT), National Taiwan University, Taipei 10617, Taiwan; 7 International Graduate Program of Molecular Science and Technology, National Taiwan University (NTU-MST), Taipei 10617, Taiwan; 8 Department of Chemical Engineering and Materials Science, Yuan Ze University, Taoyuan 32003, Taiwan; 9 Yonsei Frontier Lab, Yonsei University, Seoul 03722, Republic of Korea

**Keywords:** carboxymethyl cellulose (CMC) synthesis, cellulose production, beta-cyclodextrin, oil palm empty fruit bunch, delignification, cryogelation, hydrogel, drug delivery, tetracycline, pH controlled release, antimicrobial activity

## Abstract

Cryo-induced hydrogel from cellulose is a new class of biomaterials for drug delivery, cell delivery, bone and skin tissue engineering for cell proliferation and regeneration applications. This research aimed to synthesize cryo-induced hydrogel from cellulose and carboxymethyl cellulose (CMC) produced from empty bunch’s cell wall of *Elaeis guineensis*. First, the experiment was to produce cellulose-rich material using hot-compressed water extraction followed by alkaline delignification and bleaching with H_2_O_2_. The obtained bleached EFB cellulose was used as the substrate for CMC, and the optimal condition with the highest degree of carboxyl substitution (DS) of 0.75 was achieved when varying NaOH and monochloroacetic acid concentration as well as etherification temperature using fractional factorial design. For cryogelation study, hydrogels were synthesized from cellulose, CMC and beta-cyclodextrin (β-CD) by dissolving cellulose-based matrix in a NaOH/urea system, and the cellulose (CEL) solution was frozen spontaneously at −40 °C followed by high speed mixing to loosen cellulose fibrils. Epichlorohydrin (ECH) and Polyethylene glycol diglycidyl ether (PEGDE) were used as a cross-linker. First, the ratio of cellulose and CMC with different amounts of ECH was investigated, and subsequently the proper ratio was further studied by adding different crosslinkers and matrices, i.e., CMC and β-CD. From the result, the ECH crosslinked CMC-CEL (E-CMC-CEL) gel had the highest swelling properties of 5105% with the average pore size of lyophilized hydrogel of 300 µm. In addition, E-CMC-CEL gel had the highest loading and release capability of tetracycline in buffer solution at pH 7.4 and 3.2. At pH 7.4, tetracycline loading and release properties of E-CMC-CEL gel were 65.85 mg g^−1^ dry hydrogel and 46.48 mg g^−1^ dry hydrogel (70.6% cumulative release), respectively. However, at pH 3.2, the loading and release capabilities of Tetracycline were moderately lower at 16.25 mg g^−1^ dry hydrogel and 5.06 mg g^−1^ dry hydrogel, respectively. The findings presented that E-CMC-CEL hydrogel was a suitable material for antibiotic tetracycline drug carrying platform providing successful inhibitory effect on *Staphylococcus aureus*, *Escherichia coli* and *Pseudomonas aeruginosa*, respectively.

## 1. Introduction

Biomaterials from natural biopolymers have attracted considerable attention for broad biomedical applications such as cell delivery, drug delivery, bone and skin tissue engineering [1]. Such plant-based biopolymers include cellulose, modified cellulose, e.g., carboxymethyl cellulose (CMC), hydroxylethyl cellulose (HEC) as well as starch derivatives, e.g., cyclodextrin, have been widely used owing to their great biocompatibility, controllable solubility and reactivity from degree of substituted functional groups [2]. Biomaterials with polysaccharide constituents such as hydrogel, aerogel and cryogel have been synthesized by physical cross-linking that can lead to pores or nano-fibrillar arrangement. To improve their mechanical properties, chemical crosslinking with functionalized molecules was introduced. It has been revealed that different approaches of drying and fabrication such as vacuum drying, freeze–thawing and freeze drying can provide dissimilar biomaterials for different biomedical applications [3].

Cellulose, which is the most abundant terrestrial biopolymer, comprises numerous hydroxyl groups that facilitate high functionality for fabrication of variable hydrogels with various structural properties for advanced wound healing, drug delivery or tissue engineering. For wound healing, cellulose-based hydrogels were shaped both in sheet and granulating for filling into wound cavity for chronic wound healing [4]. Its semi-transparency offers visual accessibility to trace wound healing progress, while its high absorbency provides bacterial retention talent. Moreover, the porosity and swelling ability are imperative when dealing with negative pressure on conforming wound dressing. Apart from the biocompatibility and biodegradability, cellulose-based hydrogels provide decent humidity along with suppressing bacteria for wound dressing [5,6]. Furthermore, its three-dimensional network nature and water-retaining capacity deliver an appropriate platform for growth factors and nutrients diffusion and, alternatively, convey a context for discarding excreted biological fluids [7]. It has been reported that biological fluids and water can simply penetrate the hydrogel’s 3D structure on account of their porosity, hydrophilicity and structural consistency resemble to living tissue [8]. The additional constituents assisted for structural formation can be sodium carboxymethyl cellulose (NaCMC) [9], hydroxyethyl cellulose (HEC) [10], pectin, collagen and gelatin, as well as adhesives and elastomers [11].

The present work studied the synthesis method of nano-structured hydrogels, so-called nanogels, which are three-dimensional networks of hydrophilic, linear polymer (i.e., cellulose) or branched polymers (i.e., CMC and HEC) as well as cyclic polymer (i.e., β-cyclodextrin) connected by crosslinking agents including epichlorohydrin (ECH) or poly (ethylene glycol) diglycidyl ether (PEGDE) to improve the intact structure of nanogel obtained. ECH and PEGDE which are epoxy-functionalized molecules are well known for their reactivity toward hydroxyl groups, and are thus used for polysaccharides based material to form the ether crosslinkage [12,13,14,15]. The nanogels’ ability to absorb large quantities of water and application as a model antimicrobial drug carrier platform toward antimicrobial activity study was investigated. The influence of porosity and functionality of obtained different nanogels on pH responsive loading and controlled release of antimicrobial drug, i.e., tetracyclin was studied at different pHs (i.e., pH 3.2 and pH 7.4).

## 2. Results and Discussion

### 2.1. Cellulose Production from EFB

**Figure 1:**
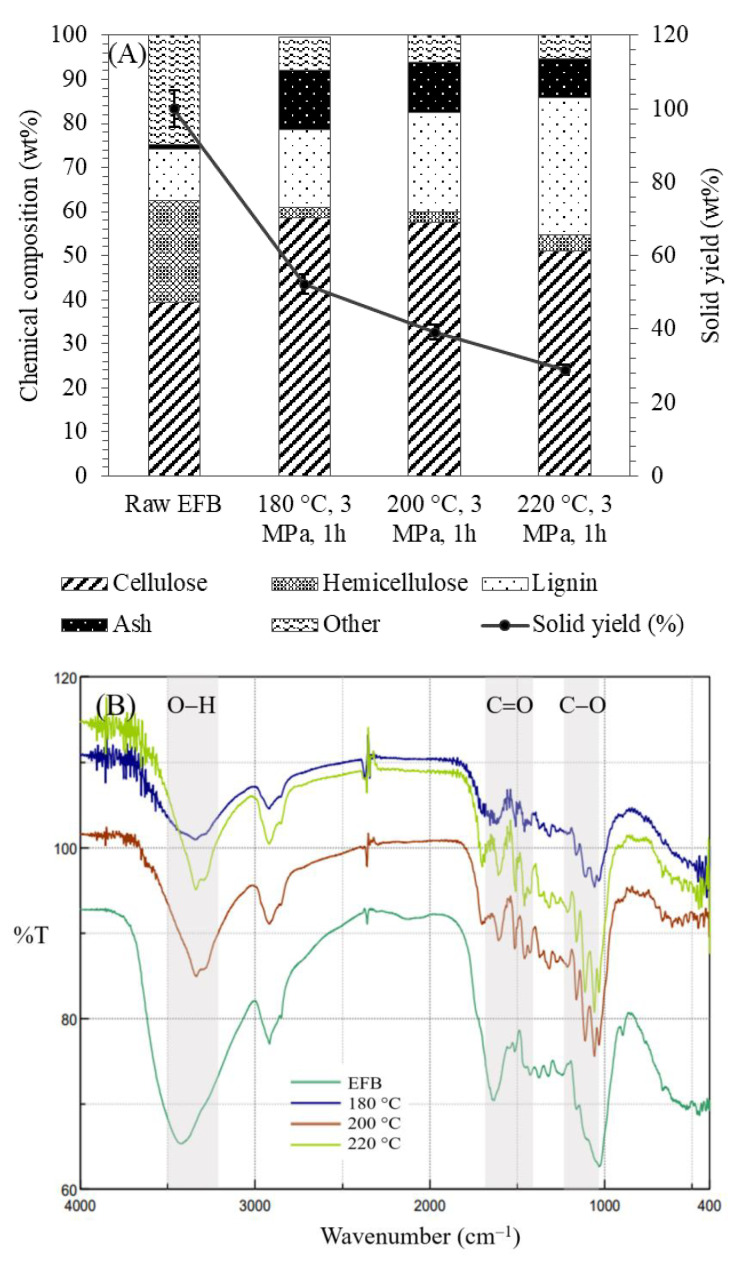
(**A**) Chemical composition, solid yield and (**B**) FTIR spectra of pretreated EFB at different hydrothermal temperatures at 180 °C, 200 °C and 220 °C for 1 h at 3 MPa.

Hydrothermal fractionation was conducted to break down EFB structure, eliminate hemicellulose and remove partial lignin constituent. At elevated temperature and pressure, the dielectric constant of water is reduced and water acts as an organic solvent at that state. It was reported that EFB fractionation at 190 °C and beyond at 3 MPa for 5–25 min, cellulose content increased substantially from 38.50% to 63–69.3% due to hemicellulose removal and partial lignin degradation [16]. In the present study, the effect of different temperatures at 180 °C, 200 °C and 220 °C for hydrothermal fractionation at 3 MPa for 1 h was investigated. As illustrated in Figure 1A, hydrothermal fractionation of EFB at 180 °C attained the highest % solid yield and that at 220 °C attained the lowest % solid yield. This was in accordance with a previous research reporting that due to the high temperature, the breakdown of lignocellulose structure of natural material is accelerated due to the solvent-like characteristic of water under elevated temperature, and pressure facilitates the dissolution of hemicellulose and lignin fractions from biomass matrix, thus loss of mass yield to solubilized hemicellulose and lignin was observed. The chemical compositional analysis of EFB and cellulose-rich fraction from EFB using hydrothermal reaction at different temperatures was performed. The findings showed that the majority of hemicellulose was removed from EFB by hydrothermal reaction in which water becomes acidic, low di-electric constant and lower viscosity closed to solvent, e.g., methanol [17]. When water is heated well above 100 °C its dielectric constant decreases and its ionic product increases. At 200 °C the dielectric constant of water is the same as that of room temperature methanol. Above 200 °C water may be an acid or base catalyst because its H_3_O^+^ and OH^−^ ion concentrations are perhaps orders of magnitude higher than in ambient water. From Figure 1A, hemicellulose removal from 23.02% hemicellulose in raw EFB to 13.51%, 11.60% and 8.83% hemicellulose which corresponds to 95.07%, 96.30% and 95.59% hemicellulose removal for 180, 200 and 220 °C hydrothermal fractionation, respectively (Table S2). In addition, it was revealed that water becomes acidic or protonated to hydronium ions (H_3_O^+^) at elevated temperature and subsequently hydronium ions act as a catalyst for the release of acetyl groups from hydrothermolysis into the liquid phase. In addition, the deacetylation of hemicellulose consequently led to generated acetic acid which acts as a catalyst for secondary hydrolysis reactions. The result was in good agreement with other hydrothermal pretreatments of EFB [16]. At the hydrothermal temperature of 180°C, the highest percentage of cellulose was obtained at 58.7% which corresponded to 77.68% cellulose recovery (Table S2); however, loss of cellulose due to cellulose degradation was observed, thus slightly lower cellulose content was found at 220 °C for 51.2% corresponding to only 37.47% cellulose recovery based on cellulose in raw EFB. This is due to the fact that at high temperature, the decay of cellulose is also accelerated. It has been reported that thermal degradation temperature under atmospheric pressure of cellulose was found at 300–400 °C, while that of hemicellulose was at 220–315 °C, and lignin was decomposed at a board range of temperature between 150 and 900 °C [18]. Recently, it has been disclosed that hydrolysis of xylooligosaccharides and partial degradation of lignin to products, i.e., sinapyl and coniferyl alcohols, were found in a hot-compressed water system when heated to 200–220 °C for 10 min at 10 MPa. In contrast, greater energy is required to breakdown cellulose structure at 250–270 °C for 30–35 min at 10 MPa [19]. As a result, hydrothermal fractionation under high pressure of 3 MPa, which was 30 times greater than atmospheric pressure, could dramatically promote hemicellulose degradation and partial lignin decomposition at temperatures lower than 220 °C. At the temperature of 200 and 220 °C for 1 h, the percentage of lignin content in the cellulose-rich fraction was in turn enhanced relatively to raw EFB. It was reported that acidic reaction condition, e.g., in the presence of H_2_SO_4_ or in this case acetic acid from deacetylation of hemicellulose, substantially impacted the degree of delignification. At elevated temperature and pressure, lignin fragment from depolymerization can apparently repolymerize onto cellulose structure and thus enhance the lignin content in a cellulose-rich material after fractionation [20]. Addition of polar solvents has been reported to increase delignification yield and the delignification could be decreased due to the apolar characteristics of the reaction solution [21] from non-polar by-products released such as monomeric phenols, guaiacol, 4-ethylphenol, 4-ethylguaiacol and syringol, which are the main products of lignin degradation [22].

As shown in Figure 1B, the functional groups of cellulose after fractionation were analyzed with FTIR. From the FTIR results, the functional groups of O–H, C=O and C–O assigned to hydroxyl group, carbonyl group and carboxylic group, respectively, were found. The peaks at 1433 and 1513 cm^−1^, attributed to aromatic structure of lignin [23,24,25,26], are prominent after hydrothermal fractionation relative to untreated EFB. This was caused by either removal or peeling of hemicellulose surrounding the lignin-cellulose matrix confirming that hydrothermal pretreatment insignificantly facilitates delignification of lignocellulosic biomass. Furthermore, the absorption bands assigned to hemicellulose moieties, i.e., the bands at 1258, 1400 and 1733 cm^−1^ [23,24,27] were disappeared or less intense due to the degradation or depolymerization of hemicellulose during hydrothermal reaction.

**Figure 2:**
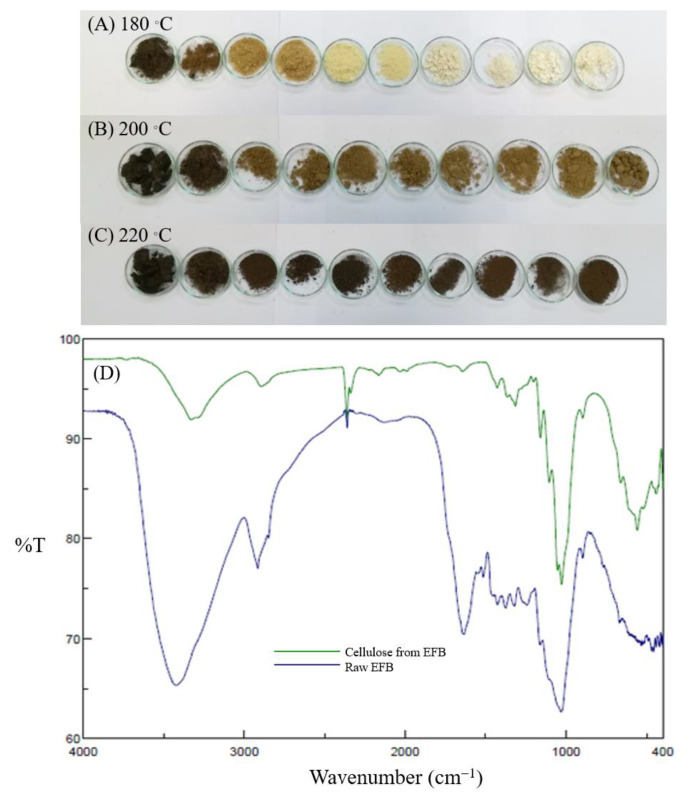
Cellulose bleaching of pretreated EFB at different hydrothermal temperatures (**A**) 180 °C, (**B**) 200 °C, (**C**) 220 °C using 30 wt% H_2_O_2_ solution at 60 °C for ten times; and (**D**) FTIR graph represent between purified cellulose from EFB using hydrothermal fractionation at 180 °C for 1 h and bleached with 30 wt% H_2_O_2_ for seven times at 60 °C compared with raw EFB.

To enhance cellulose purity by eliminating residual hemicellulose and lignin, repeated bleaching of extracted cellulose from EFB was conducted using hydrogen peroxide at 60 °C. As demonstrated in Figure 2A, the color of bleached cellulose was brighter compared with unbleached ones for all hydrothermal temperatures applied. The cellulose that was produced from hydrothermal reaction at the temperature of 180 °C dynamically changed its color and became whiter at only the 5th to 7th bleachings. While cellulose extracted from the temperatures of 200 and 220 °C turned white, it was at a much slower rate than that at 180 °C. The reason was mainly due to the darker color of lignin chromophore from the repolymerization of the lignin fragment from the depolymerization process when heating at 200 and 220 °C. It was reported that the main lignin chromophore found in lignocellulose was 2,5-dihydroxy-[1,4]-benzoquinone (DHBQ), and a minor component, 2,5-dihydroxyacetophenone (DHA), while hexeneuronic acids (HexA) were found to cause discoloration (yellowing/brightness reversion) in cellulose pulps from xylan-containing biomass [28]. We adopted the L × a × b color measurement approach [29], as shown in Table S3. The whiteness of commercial cellulose was 51.8, whereas the purified cellulose from EFB that was processed with 5 consecutive bleaching had the whiteness value at 41.6 and after 7 consecutive bleaching, the whiteness of purified cellulose from EFB was increased to 52.9 which was higher than commercial cellulose. Extracted cellulose from EFB at 200 and 220 °C remained brownish (whiteness = 39.8) and dark brownish (whiteness = 26.9), respectively, at even the 10th time of bleaching. Consequently, when the energy input for hydrothermal process and chemical usage in bleaching process that impact the cost of production were taken into account, the purified cellulose from 180 °C hydrothermal reaction at 3 MPa for 1 h was selected as precursor for semi-pilot scale production of cellulose for cryo-induced cellulose-based hydrogel formation.

FTIR spectra of purified cellulose from 180 °C, 3 MPa, 1 h plus 7th bleaching and raw EFB are shown in Figure 2B. The peak intensity of purified cellulose from EFB was clearly seen at the wavenumber of 2400 cm^−1^ which represented carboxylic functional group [30], while less intensity was found in raw EFB. The FTIR peaks at the wavenumber of 1515 cm^−1^, which represents a benzene ring of lignin [10], and at the wavenumber of 1240 cm^−1^, which represents the carbonyl bond of lignin can be found in EFB but not in the purified cellulose from EFB. This clearly revealed that lignin has been extracted out from the hydrothermal fractionation and subsequent bleaching process due to the chemical oxidation reaction of hydrogen peroxide. It was reported that alkaline hydrogen peroxide dissociates oxygen with free electrons and radicals [31] which interact with residual hemicellulose, lignin and chromophores [32]. This process reduces the chromophore molecular size, and additionally decomposes chromophore, thus led the cellulose color alteration to paler and brighter during bleaching.

### 2.2. CMC Synthesis from EFB Derived Cellulose

Carboxymethyl cellulose was synthesized from purified cellulose from EFB as depicted in Figure S1. Degree of substitution (DS) and whiteness of synthesized CMC from EFB derived cellulose at different CMC synthesis conditions (%NaOH, %monochloroacetic acid and etherification temperature) were presented in Table S3. As shown in Scheme S1, the first step of CMC synthesis is cellulose mercerization or alkalization in NaOH solution. In this step, cellulose is protonated at the CH_2_OH functional group, and subsequently substituted by Na^+^ from NaOH to form CH_2_ONa functional group. The position of –ONa group at each anhydrous glucose unit (AGU) will be further replaced with carboxymethyl group in second step of etherification. The presence of inert C_1_–C_4_ alcohols, i.e., ethanol [33], 2-propanol [34], isopropanol [35] and isobutyl alcohol [36], as a diluent in mercerization step could enhance cellulose swelling and penetration of NaOH into cellulose molecules [37].

**Figure 3:**
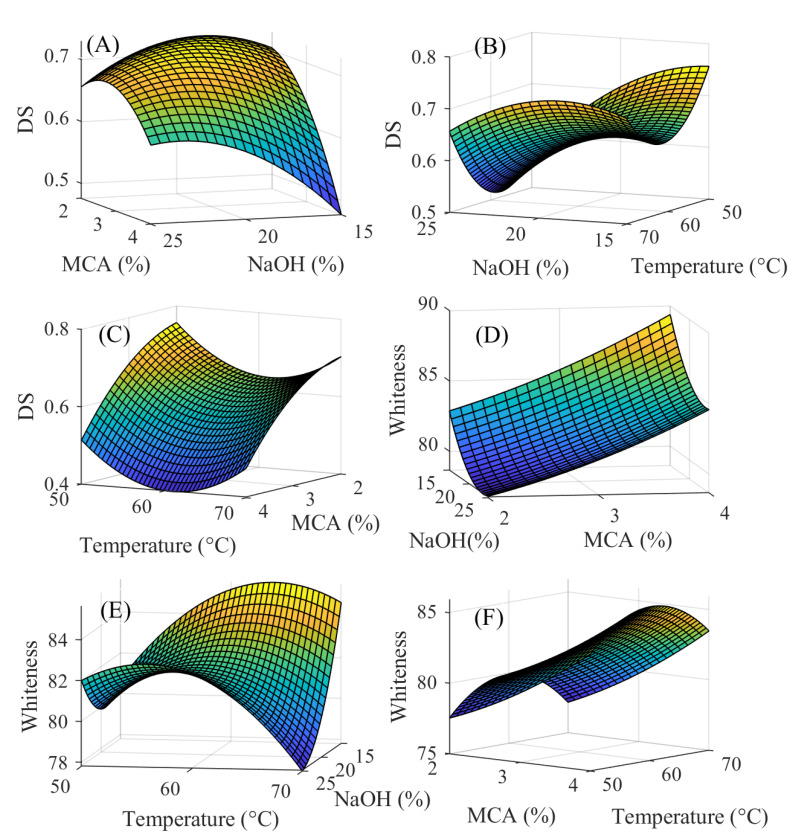
Three dimensional surface plots of quadratic regression models for (**A**–**C**) degree of substitution (DS) and (**D**–**F**) whiteness of CMC synthesized from purified cellulose when NaOH concentration (15–25 wt%), MCA concentration (2–4 wt%) and etherification temperature (50–70 °C) were varied.

In the second step of etherification using monochloroacetic acid, –ONa group of each AGU was substituted by –OCH_2_COONa so called carboxymehtyl group which represents degree of substitution toward AGU and resulted in NaCl byproduct as shown in Scheme S1. From the results (Table S3), DS values varied according to NaOH concentration (wt%), monochloroacetic acid concentration and etherifying temperature. Under the optimized conditions, the DS of CMC was achieved at 0.76 from 15 wt% NaOH at room temperature (30 °C) for 30 min. Afterward, etherification with 2% *w*/*v* monochloroacetic acid at 50 °C for 3 h. From ANOVA of quadratic regression of DS (Table S4), the 3D surface plots of DS regression model according to variation of NaOH, monochloroacetic acid concentration and etherifying temperatures (DS = 4.49771 − 0.12675x_3_ + 0.00875x_1_x_2_ + 0.000725x_1_x_3_ + 0.000375x_2_x_3_ − 0.00183x_1_^2^ − 0.04521x_2_^2^ + 0.000938x_3_^2^ with R^2^ = 0.81) were shown in Figure 3A–C. The finding demonstrated that an increase in NaOH concentration resulted in DS decrease. Even though there were interaction of NaOH, monochloroacetic acid concentration and temperature, it was apparent that an increase in monochloroacetic acid concentration from 2 to 4% *w*/*v* and an increase in etherification temperature from 50 to 70 °C led to a decline in DS. The decrease in DS due to an increase in etherification temperature was in good agreement with previous work studied between 30 °C and 70 °C for CMC synthesis from sugarcane bagasse. Based on it, the optimum condition of NaOH concentration of 28.4%, MCA mass of 1.14 g, temperature of 57.85 °C and reaction time of 4.01 h, which the CMC had, and the DS of 1.085 [30]. Therefore, an optimization of all parameters is necessary for a different terrestrial cellulosic precursor. It was revealed that a significant enhancement in the DS value while increasing dosage of etherification agent (i.e., MCA) was obtained to some certain value at the ratio of NaMCA:AGU of 2:1, afterward an increase in MCA could lead to the decline in DS [38]. However, in the present study, an optimal ratio of MCA:AGU was 1:1.65, corresponding to 10 g MCA:15 g purified cellulose with an anhydrous glucose unit (MW = 162 g mol^−1^) = 0.90 × cellulose weight (g). Nevertheless, the redundant chemicals in the system, such as NaCl, could unfavorably react with MCA then react with excess NaOH in the system and thus generate a by–product (sodium glycolate) from the following reaction [NaOH + Cl–CH_2_COONa → OH–CH_2_COONa + NaCl], and consequently reduce the rate of carboxymethylation reactions which are the main reactions [37]. It has been additionally disclosed that surplus NaOH will eliminate some cellulose chains resulting in a decrease in DS [39].

In contrast to DS, an increase in MCA concentration from 2 to 4%w/v and etherifying temperature from 50 to approximately 65 °C substantially enhanced the whiteness of CMC, as demonstrated in Table S5 for ANOVA of the quadratic regression for whiteness (whiteness = −33.375 + 3.80625x_3_ − 0.035x_1_x_2_ − 0.041x_1_x_3_ + 0.0125x_2_x_3_ + 0.057125x_1_^2^ + 0.495833x_2_^2^ − 0.0524x_3_^2^ with R^2^ = 0.91), and Figure 3D–F for 3D surface plots of CMC whiteness from the model. The optimal whiteness value achieved was 89.37 at the mercerization reaction condition of 15 wt% NaOH, and etherification at 4% *w*/*v* MCA at 64 °C. Based on high DS and whiteness of CMC with the lowest yellowness (Figure S1), as well as low chemical usage, the synthesis condition of 15 wt% NaOH and 2% *w*/*v* MCA (or 10 g MCA) at 70 °C with high DS of 0.70 and whiteness of 81.88 was calculated from the model, which corresponded to the CMC3 sample with DS of 0.75 ± 0.01 and ΔE of 80.8 (Table S3 and Figure S1), and was selected for cryogelation of cellulose and CMC.

### 2.3. Cryogelation of Purified Cellulose and CMC from Elaeis guineensis Using Epichlorohydrin as a Crosslink Agent

**Figure 4:**
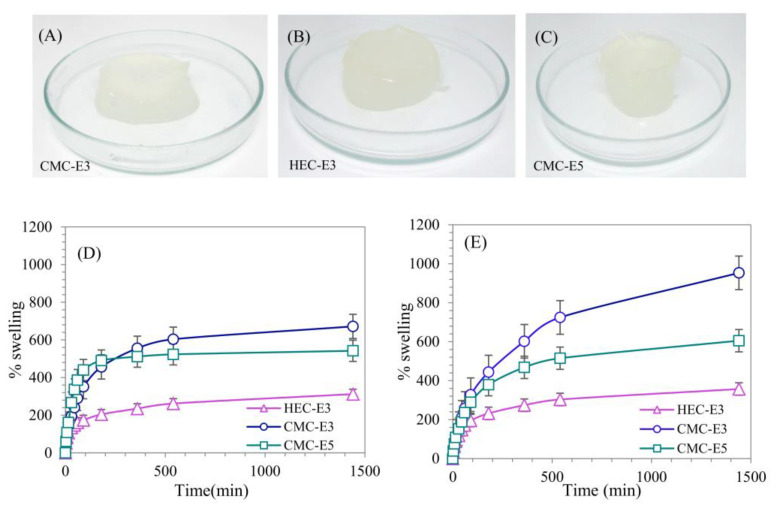

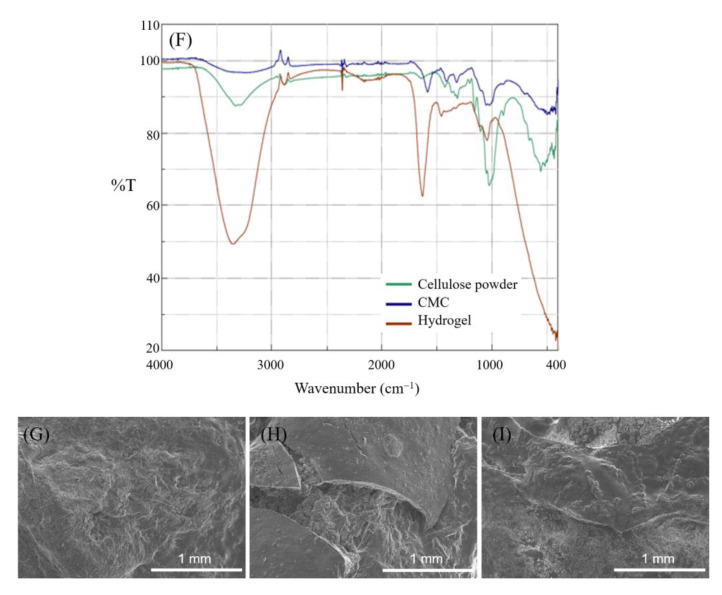
(**A**) Hydrogel CMC-E3, (**B**) Hydrogel HEC-E3, (**C**) Hydrogel CMC-E5 and swelling percentage of the hydrogels (**D**) at 25 °C for 24 h, and (**E**) at 35 °C for 24 h; (**F**) FTIR spectra of CMC-E3 hydrogel, CMC and cellulose powder; scanning electron microscopic images of hydrogels (**G**) CMC-E3, (**H**) HEC-E3 and (**I**) CMC-E5.

For the synthesis of hydrogel using the cryogelation technique in which the cellulose structure was broken down by icy crystals during rapid freezing at −20 °C, the addition of carboxymethyl cellulose (CMC) or hydroxyethyl cellulose (HEC) or cassava starch to enhance efficient networking and water holding capacity was studied. Moreover, the impact of different amounts of epichlorohydrin (ECH) as a crosslinker was investigated as shown in Table S6. From the result of hydrogel synthesis, there were three examples that can be formed as hydrogel, namely CMC-E3, CMC-E5 and HEC-E3; the appearance of hydrogels is shown in Figure 4A–C, respectively. Each hydrogel fabricated by cryo-induced technique was tested for its swelling ratio when immerged in distilled water at 25 °C and 35 °C for 24 h as demonstrated in Figure 4D,E, respectively. When all three hydrogels were tested for water holding capacity at 25 °C, hydrogels had different percentages of swelling ratio particularly after 90 min of absorption. In the first phase within 90 min, all three hydrogels had a similar percentage of swelling. However, over time until the end of the experiment (1440 min or 24 h), the CMC-E3 hydrogel had the highest swelling ratio percentage of 670% followed by CMC-E5 hydrogel at 540%, and HEC-E3 hydrogel at 310%, respectively. An increase in absorption temperature could considerably enhance water swelling ratio. When all three hydrogels were tested at 35 °C, the CMC-E3 hydrogel had greater percentage of swelling at 950%, followed by CMC-E5 with swelling percentage of 600% and HEC-E3, with swelling ratio of 350% relative to swelling ration at 25 °C. Therefore, CMC-E3 hydrogel showed the most favorable hydrogel with highest water holding capacity and was used for subsequent study on optimization for drug delivery platform. 

As demonstrated in Figure 4F, functional groups of hydrogel CMC-E3, which exhibited very good appearance and swelling properties, were identified using FTIR spectroscopic analysis compared with spectra of CMC and purified cellulose powder from EFB. The peak at 3300–3500 cm^−1^, which belongs to the hydroxyl group, was prominent in CMC-E3 hydrogel owing to the large amount of hydroxyl groups. This peak additionally revealed the presence of bound water in the structure of hydrogel which was different to cellulose powder and carboxymethyl cellulose. In contrast, the cellulose constituent is water-insoluble, as a result, the peaks at 3300–3500 cm^−1^ was found very low intensity due to lower amount of hydroxyl groups, on the one hand, intra-cellular hydrogen bonds between cellulose chain made hydroxyl group invisibly appeared in FTIR spectra. When the CMC-E3 hydrogel was formed, the functional groups were additionally compared before and after water absorption, and a substantial greater peak intensity of hydroxyl functional group appeared (Figure 4F). In addition to functional groups comparing using FTIR, the physical properties of the vacuum-dried hydrogel samples were able to be analyzed by scanning electron microscopy (SEM) as illustrated in Figure 4G–I, representing CMC-E3, HEC-E3 and CMC-E5 hydrogels, respectively. Electron microscopy revealed that at 50× magnification, no hydrogel porosity was observed in the hydrogel vacuum drying step. It has been reported that drying technique meant substantially toward porosity of hydrogel. Changing phase of water from liquid to gas under vacuum drying may cause pore collapse comparing to freeze drying in which icy crystal of water in solid phase was sublimed to vapor spontaneously, therefore superior porosity of material was obtained [40].

### 2.4. Effect of Cellulose-to-CMC Ratio with ECH Crosslinker on Hydrogel Properties

**Figure 5:**
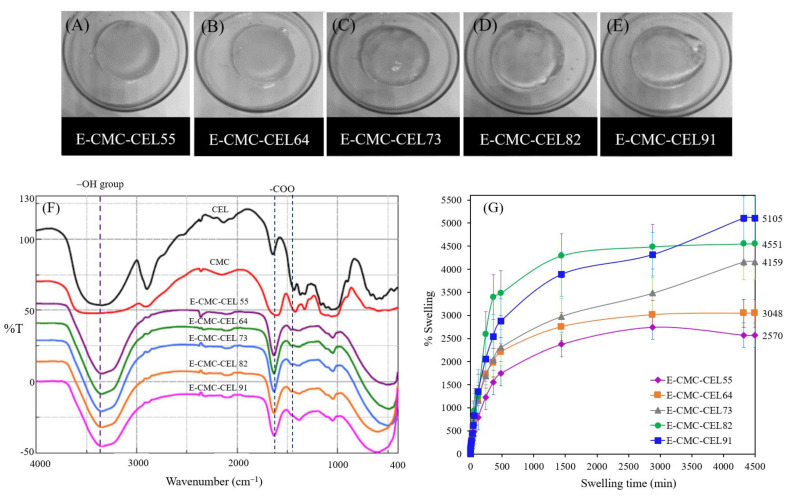
The shape of E-CMC-CEL gels with ECH as a crosslinker; (**A**) E-CMC-CEL55, (**B**) E-CMC-CEL64, (**C**) E-CMC-CEL73, (**D**) E-CMC-CEL82, (**E**) E-CMC-CEL91, (**F**) FT-IR spectra of E-CMC-CEL gels by using ECH as a cross linker and (**G**) %swelling of CMC-CEL gels with ECH cross linker in distilled water at 25 °C for 72 h.

Synthesis of CMC-CEL hydrogels by varying CMC-to-cellulose ratios using ECH as a crosslinker was investigated. With the proportion of the substrate as shown in Table S1, five formulas of CMC-CEL gels were synthesized as follows: E-CMC-CEL55, E-CMC-CEL64, E-CMC-CEL73, E-CMC-CEL82 and E-CMC-CEL91. Each type had a different composition of cellulose and CMC in synthesis at a constant amount of 3 mL ECH. The appearance of CMC-CEL hydrogels at different CMC soln: Cell soln ratios was shown in Figure 5A–E. From the observation, E-CMC-CEL91 in which CMC soln:Cell soln was 9:1 (*w*/*w*) exhibited more liquid-like hydrogel compared with other formulas with less amount of CMC constituent. It was found that a decrease in CMC ratio could enhance hardness of hydrogel, and thus the E-CMC-CEL55 showed the utmost rigid shape among E-CMC-CEL hydrogels. The rheological properties of hydrogel were consecutively studied to confirm the characteristic of hydrogels.

The functional groups of CMC-CEL hydrogels with ECH crosslinker were demonstrated through FTIR spectra at wave number in a range of 400–4000 cm^−1^, as shown in Figure 5F. The functional group of all E-CMC-CEL hydrogels revealed the hydroxyl functional group from cellulose backbones at 3356 cm^−1^ and the carboxyl functional group from CMC networks at 1700 and 1420 cm^−1^. It has been reported that the hydroxyl functional group in the hydrogel makes the hydrogel structure stronger and the carboxyl functional group would help the hydrogels to absorb water better due to the electrostatic interaction force [12]. From the 1700 and 1420 cm^−1^ wavenumbers, E-CMC-CEL91 was found to have the highest amount of carboxyl groups. Therefore, E-CMC-CEL91 presumably displayed a superior chemical structure for water holding material among other formulas tested. 

The study on water swelling of E-CMC-CEL hydrogels with ECH cross-linker in distilled water at room temperature (25 °C) lasted for 3 days as shown in Figure 5G. The relationship between swelling time and percentage of swelling ratio of hydrogels was monitored. The results exhibited that E-CMC-CEL91 had the highest swelling ratio percentage of 5105%, while E-CMC-CEL55 had the lowest swelling ratio percentage of 2571%. The swelling ratio of E-CMC-CEL gels depends greatly on amount of CMC constituent. E-CMC-CEL91 had the greatest amount of CMC component than other formulas, therefore E-CMC-CEL91 had more hydroxyl group which could enlarge hydrogel networks by electrostatic repulsions force than other hydrogels. The increase in swelling ratio can be attributed to the fact that the carboxyl groups are highly ionized in a neutral pH environment. This gives rise to the hydrophobic-to-hydrophilic transition of the hydrogel backbones and electrostatic repulsion between the negatively charged chains [41]. When comparing the percentage of swelling ratio of E-CMC-CEL gels with previous work, it was found that the synthetic CMC/CELL hydrogels had 1000 g g^−1^ swelling ratio of wet gel weight:dry gel weight which corresponded to a 100,000% swelling ratio [12]. The lower swelling ratio in the present work (5105%) compared with previous work was mainly due to the difference in size of hydrogel particles and the method of drying as well as the variation of vacuum drying condition which could reduce bound water, apart from free water, within hydrogel structure, that prominently influenced the water holding capacity of hydrogel from dry state to fully wet state.

**Figure 6:**
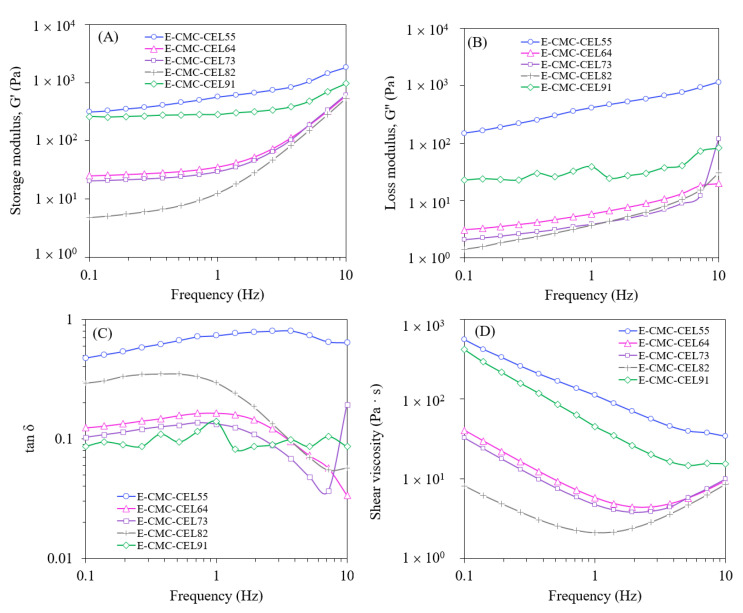
Rheological properties of cellulose hydrogel. (**A**) Storage modulus, (**B**) loss modulus, (**C**) tan δ and (**D**) shear viscosity of swelling gels.

Rheological characteristics of E-CMC-CELL hydrogels were analyzed using a sweep test with a varied frequency of rheometer, as demonstrated in Figure 6. The rheological moduli, tan δ and viscosity of the ECH crosslinked cellulose/CMC hydrogels exhibited different tendencies during the angular frequency sweeps. As shown in Figure 6A, the storage modulus (G′) of all hydrogels in the study was always higher than the loss modulus (G″) (Figure 6B), and there were no cross-over points beyond tan δ = 1 for any of the hydrogels (Figure 6C). This indicated that the hydrogels had the gel-like characteristic structure and additionally zero shear viscosity was not detected [42]. Moreover, tan δ values illustrated in Figure 6C was in the range 0.03–0.8, representing that the elastic properties were superior to the viscous properties in the dynamic viscoelastic behavior [43] of the ECH crosslinked CMC/Cellulose hydrogels for all CMC-to-cellulose ratios. As seen in Figure 6A, the G′ of E-CMC-CEL55 and E-CMC-CEL91 exhibited almost no dependence on the frequency characteristic of a permanent gel network. In the case of E-CMC-CEL64 and E-CMC-CEL73, the G′ value decreased by about 1 order of magnitude, and even decreased by about two orders of magnitude for E-CMC-CEL82. Moreover, G′ of E-CMC-CEL64, E-CMC-CEL73 and E-CMC-CEL82 showed drastic changes in the frequency dependence over 1 rad s^−1^, demonstrating that the aforementioned hydrogels possessed the viscoelastic behavior of a weak gel [42]. From these observations, it could be clarified that the viscoelastic behaviors of gels strongly depended on the CMC-to-cellulose ratio of the hydrogels, and that the addition of larger amount of cellulose based on CMC (E-CMC-CEL55 from CMC soln:Cell soln = 5:5) caused a significant increase in solidified gel, the moduli (G′ and G″) and viscosity. The findings suggested that more solid-like hydrogel such as E-CMC-CEL55 is applicable to use as a sheet form for wound dressing while more liquid-like hydrogel such as E-CMC-CEL91 is possible as a filler into wound cavity for chronic wound healing.

### 2.5. Influence of β-Cyclodextrin as Networking Precursor in Cryo-Induced Cellulose Hydrogel

**Scheme 1:**
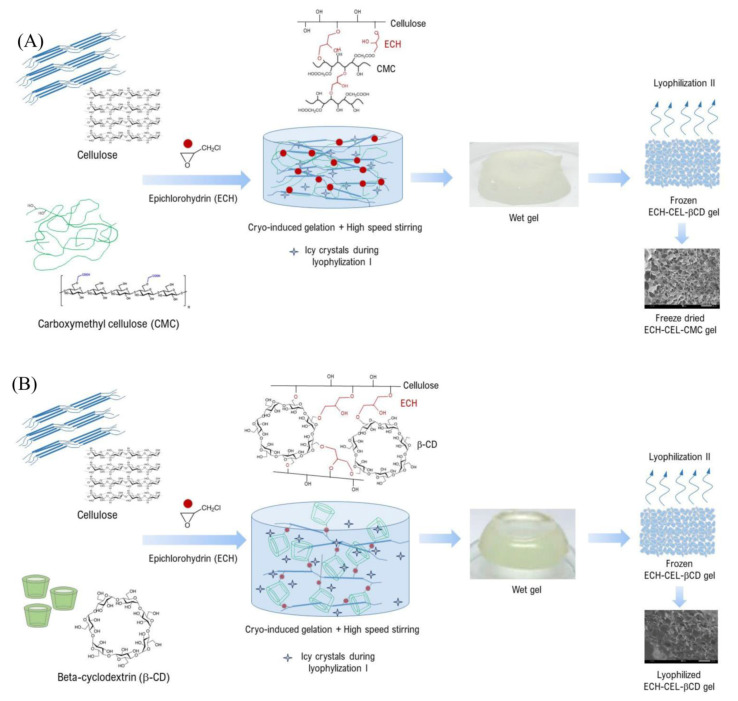
Cryo-Induced Cellulose-based Nanogel from *Elaeis guineensis*; (**A**) Epichlorohydrin crosslinked cellulose-CMC (E-CMC-CEL) hydrogel and (**B**) Epichlorohydrin crosslinked cellulose-β-cyclodextrin (E-CEL-βCD) hydrogel.

**Figure 7:**
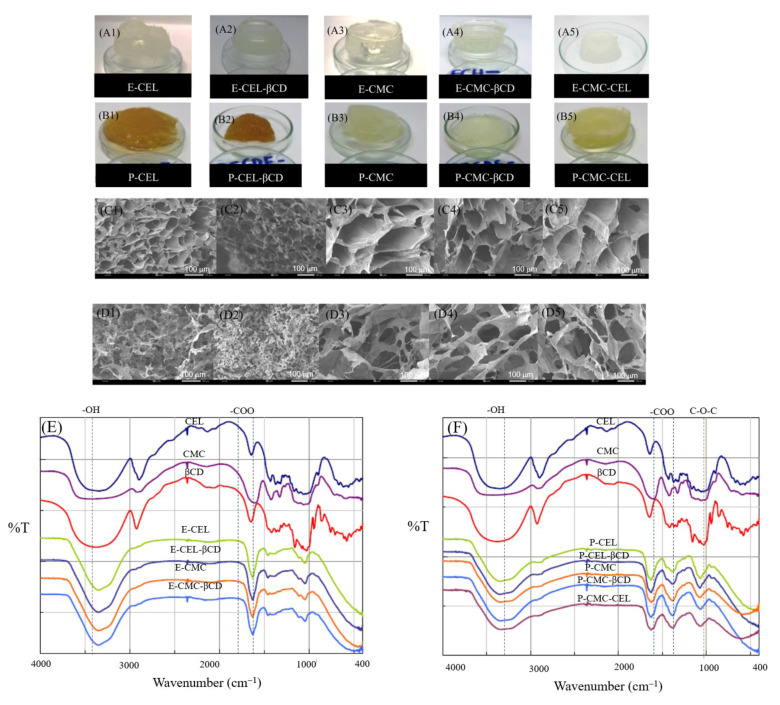
(**A**) The shape of (**A1**) E-CEL, (**A2**) E-CEL-βCD, (**A3**) E-CMC, (**A4**) E-CMC-βCD, and (**A5**) E-CMC-CEL gels with ECH as a cross linker; (**B**) The appearance of (**B1**) P-CEL, (**B2**) P-CEL-βCD, (**B3**) P-CMC, (**B4**) P-CMC-βCD and (**B5**) P-CMC-CEL gels with PEGDE as a cross linker; and SEM images at ×200 magnification of (**C**) epichlorohydrin crosslinked hydrogels namely (**C1**) E-CEL, (**C2**) E-CEL-βCD, (**C3**) E-CMC, (**C4**) E-CMC-βCD and (**C5**) E-CMC-CEL and (**D**) PEGDE crosslinked hydrogels namely (**D1**) P-CEL, (**D2**) P-CEL-βCD, (**D3**) P-CMC, (**D4**) P-CMC βCD and (**D5**) P-CMC-CEL; FT-IR spectra of CEL, CEL-βCD, CMC, CMC-βCD and CMC-CEL gels by using (**E**) ECH as a cross linker and (**F**) PEGDE as a cross linker.

The influence of the replacement of cellulose and CMC with βCD when using ECH cross linker was additionally investigated. The synthesis of E-CEL, E-CEL-βCD, E-CMC and E-CMC-βCD gels was performed with the ratio of CEL or CMC ratio to βCD for CEL–βCD and CMC-βCD gels of 9:1 (the best condition of E-CMC-CEL). At this condition, hydrogels namely E-CEL, E-CEL-βCD, E-CMC and E-CMC-βCD were successfully formed, as shown in Figure 7A. E-CEL and E-CEL-βCD hydrogels, in which cellulose was used as a major component in the synthesis, were slightly turbid and the hardness of the hydrogels was greater than that of E-CMC and E-CMC-βCD hydrogels using CMC as a matrix. This was mainly because ECH contains two functional groups, an epoxide and alkyl chloride, therefore ECH can undergo many chemical reactions after hydrolysis in the presence of NaOH [44]. It was apparent that hydroxyl and methyl groups in CMC could facilitate the bonding between epoxide and alkyl chloride groups of ECH towards hydroxyl groups of either cellulose (Scheme 1A) or βCD along with the molecular structure of CMC (Scheme 1B) which led to more flexible hydrogel fabricated.

Apart from ECH, the effect of PEGDE crosslinker on synthesis CEL, CEL-βCD, CMC, CMC-βCD and CMC-CEL hydrogels was studied. As illustrated in Figure 7B, synthesis of CEL, CEL-βCD, CMC, CMC-βCD and CMC-CEL hydrogels using PEGDE as a crosslinker with the ratio of CEL or CMC ratio to βCD was 9:1 (the best condition of E-CMC-CEL) was successfully performed, especially for P-CMC, P-CMC-βCD and P-CMC-CEL hydrogels, as shown in Figure 7B. In the case of the cellulose matrix, it was found that PEGDE was not a suitable crosslinker for the P-CEL and P-CEL-βCD hydrogel formation. Apart from that, hydrogels with PEGDE crosslinker were more liquefied than hydrogels with ECH cross linker. Moreover, P-CEL and P-CEL-βCD hydrogels, which had cellulose as the main component, exhibited a darker color than P-CMC, P-CMC-βCD and P-CMC-CEL, in which CMC was the major constituent.

SEM image analysis of all hydrogels with ECH and PEGDE crosslink agents was performed as demonstrated in Figure 7C,D, respectively. Prior to SEM measurement, CEL, CEL-βCD, CMC, CMC-βCD and CMC-CEL gels with ECH and PEGDE crosslinkers were swelled in water for 3 days, and subsequently underwent freeze drying. From Figure 7C, the average pore size of the hydrogels was ranged as E-CMC-CEL (240–360 µm) > E-CMC (160–280 µm) > E-CMC-βCD (140–200 µm) > E-CEL = E-CEL-βCD (80–100 µm). Expansion of pore size after water absorption came primarily from the carboxyl functional group from CMC molecules which generated electrostatic repulsions force caused the enlarged pore size of CMC containing hydrogels. The results were in good agreement with previous research, which stated that the size of the pore in a hydrogel structure depends on the amount of CMC that is composed of intermolecular carboxyl group structure due to electrostatic repulsions [14]. From Figure 7D, similar consequence was obtained, and the average pore size of CMC containing hydrogels was much greater than cellulose and βCD hydrogels as follows; P-CMC (140–220 µm) > P-CMC-βCD (140–160 µm) > P-CMC-CEL (120–200 µm) > P-CEL = P-CEL-βCD (12–40 µm). 

The functional groups of CEL, CEL-βCD, CMC, CMC-βCD and CMC-CEL hydrogels with ECH and PEGDE crosslinkers were analyzed by FTIR spectroscopy at wave number between 400 and 4000 cm^−1^ as shown in Figure 7E,F, respectively. From Figure 7E,F, hydroxyl functional group at the wavenumber 3300–3500 cm^−1^ was found. A carboxylate functional group at 1700 and 1420 cm^−1^ and an ether group at 1051 cm^−1^ were also detected. Hydrogels which had cellulose as the main component (E-CEL, E-CEL-βCD, P-CEL and P-CEL-βCD) exhibited higher intensity of hydroxyl group that could enhance the strength of hydrogel structure, nevertheless the decrease in swelling properties was observed. Hydrogels which had CMC as the core constituent (E-CEL, E-CEL-βCD, E-CMC-CEL, P-CEL, P-CEL-βCD and P-CMC-CEL) had more carboxylate group that could consecutively enhance swelling properties of hydrogels. Comparison of FTIR peaks between Figure 7E,F from hydrogels with ECH and PEGDE crosslinkers, respectively, demonstrated more carboxyl groups from ECH crosslinker than that from PEGDE crosslinker; however, fewer ether groups were found in ECH crosslinker compared with PEGDE crosslinker. Although hydrogels with ECH crosslinker had less ether group, on the other hand, the carboxyl group had more electrostatic repulsion force than the ether group. The negative charge present on the pendant carboxyl group after deprotonation participates in the electrostatic repulsion that initiates the swelling behavior [45]. Therefore, the swelling property of hydrogels with the ECH crosslinker was presumably superior to the hydrogels with a PEGDE crosslinker. 

**Figure 8:**
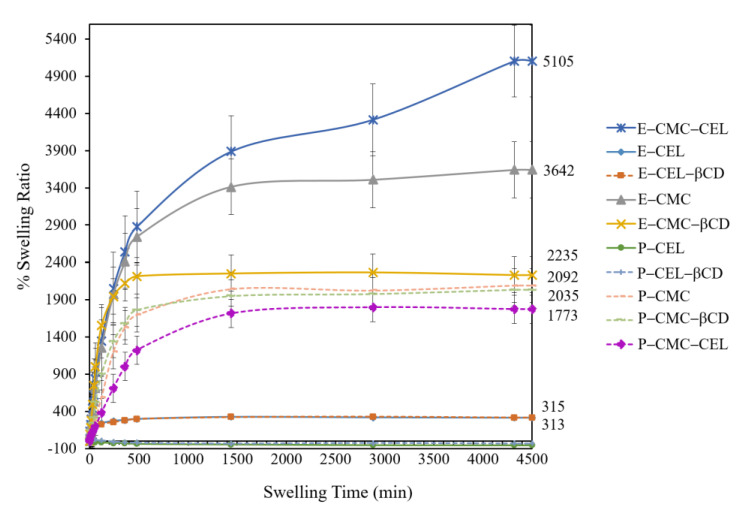
Swelling properties of CEL, CEL−βCD, CMC, CMC-βCD and CMC−CEL gels with ECH and PEGDE cross linkers in distilled water.

The aforementioned statement, therefore, was confirmed by the swelling study of CMC-CELL and β-CD with ECH and PEGDE crosslinkers as shown in Figure 8. In this study, the CEL, CEL-βCD, CMC, CMC- βCD and CMC-CEL hydrogels with ECH and PEGDE crosslinkers were swelled in distilled water at room temperature for 3 days. The alteration of swelling ratio during the time course of absorption was monitored. From Figure 8, swelling ratio of hydrogel E-CMC-CEL had the highest swelling 5105% followed by E-CMC with 3642% swelling ratio, while P-CEL and P-CEL- βCD hydrogels had the lowest swelling ratio at −33% and −58%, respectively. When comparing the swelling capacity of various types of hydrogels, the result was E-CMC-CEL > E-CMC > E-CMC-Βcd > P-CMC > P-CMC-βCD > P-CMC-CEL > E-CEL-βCD > E-CEL > P-CEL = P-CEL-βCD. The results suggested that smallest pore size hydrogels, i.e., P-CEL and P-CEL- βCD (12–40 µm) as well as E-CEL = E- CEL-βCD (80–100 µm) exhibited lowest water holding capacity. The findings were additionally inferred that these materials are possibly used for other biomedical applications such as bone and skin regenerative scaffold or tissue engineering instead of wound dressing purpose. 

**Figure 9:**
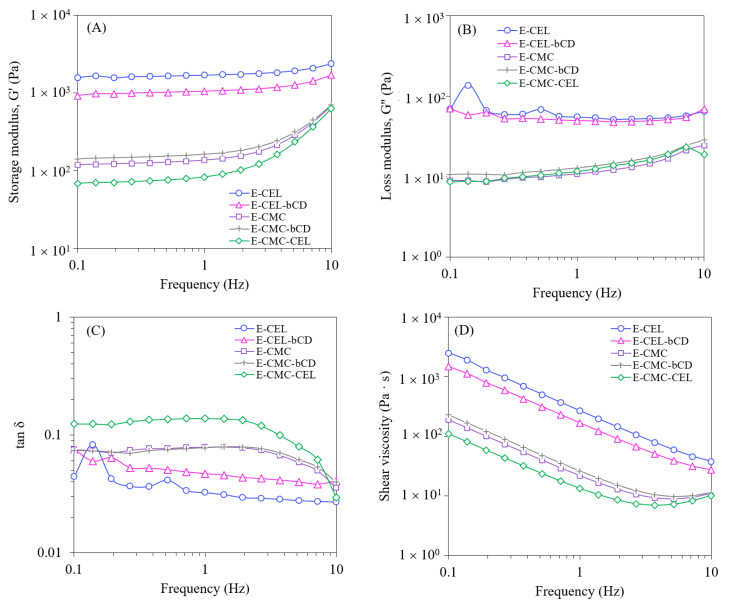
Rheological properties of ECH crosslinked cellulose, CMC, βCD-based hydrogel (**A**) Storage modulus, (**B**) Loss modulus, (**C**) tan δ and (**D**) shear viscosity of swelling gels.

**Figure 10:**
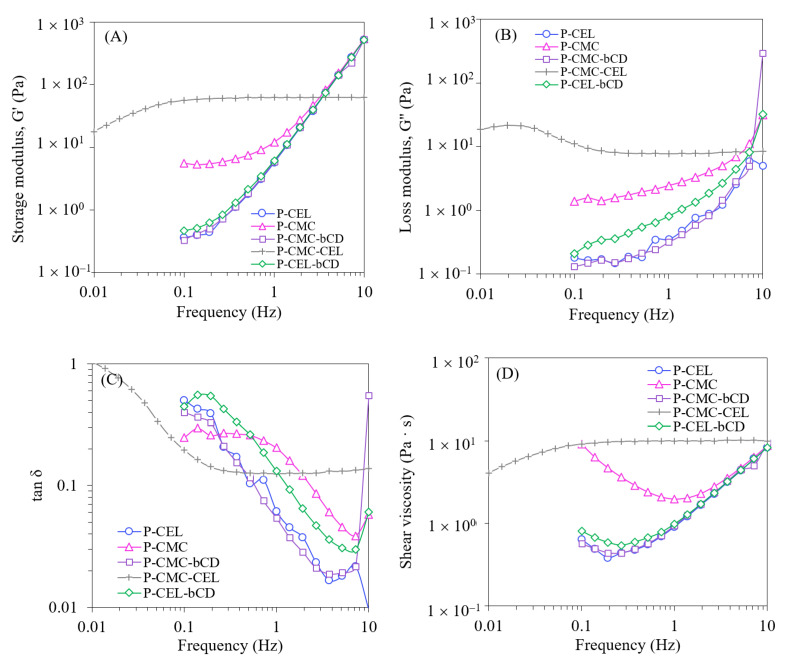
Rheological properties of PEGDE crosslinked cellulose, CMC, βCD-based hydrogel (**A**) Storage modulus, (**B**) Loss modulus, (**C**) tan δ and (**D**) shear viscosity of swelling gels.

Rheological analysis of ECH and PEGDE crosslinked cellulose, CMC and β-CD was investigated, as shown in Figure 9 and Figure 10, respectively. The storage modulus (G′), loss modulus (G″), tan δ and shear viscosity of swelling gels were measured. From Figure 9A, E-CEL and E-CEL-βCD showed only slightly change in G′ when changing frequency from 0.1 to 10 rad s^−1^ which meant that both hydrogels were strong hydrogel while drastic change in G′ of E-CMC-CEL > E-CMC > E-CMC-βCD, respectively, from 1 to 10 rad s^−1^ indicating high viscoelastic behavior of hydrogels, especially E-CMC-CEL. A similar tendency was found for G″ (Figure 9B) and shear viscosity (Figure 9D) with increasing frequency. In terms of tan δ, a range of 0.02 and 0.2 (Figure 9C) for all ECH crosslinked hydrogels was obtained representing the more elastic than viscous characteristic gels. Nevertheless, PEGDE crosslinked hydrogels as demonstrated in Figure 10 showed greater dependency of the change in G′ (Figure 10A) and G″ (Figure 10B) as well as shear viscosity (Figure 10D) on a frequency from 0.1 to 10 rad s^−1^, indicating softer or liquid-like hydrogel behavior of P-CMC-βCD > P-CEL > P-CEL-βCD > P-CMC > P-CMC-CEL. P-CMC-CEL was found the strongest hydrogel with only slightly change in G′, G″ and shear viscosity on frequency. The tan δ was in a range of 0.01 and 1.0 (Figure 10C) for most of PEGDE crosslinked hydrogels i.e., P-CMC-βCD, P-CEL, P-CEL-βCD and P-CMC signified it was more viscous than elastic characteristic gels. Therefore, it can presumably conclude that PEGDE crosslinked hydrogels were more suitable for the application of filling hydrogel in wound healing scenario rather than sheet forming hydrogel.

### 2.6. pH Responsive Antimicrobial Drug Loading and Release of Hydrogel

Tetracycline (TC) is antibiotic used for acne, cholera, brucellosis, plague, malaria and syphilis treatment. For TC loading study, all 10 types of hydrogels were tested in various pH conditions including base condition (pH 7.4) and acid condition (pH 3.2) using 0.25 mg mL^−1^ initial TC concentration at 25 °C for 3 days. The functional groups of TC in hydrogel were analyzed by FT-IR spectral analysis of loaded TC hydrogels, as shown in Figure S2. When compared with FTIR spectra of neat hydrogels (Figure 7E,F), it was found that the carboxyl and ether functional groups of hydrogels after TC loading were considerably reduced. This was mainly because the carboxyl and ether groups can create complex bonds with TC drugs, and thus TC was apparently upholding inside the hydrogels.

**Figure 11:**
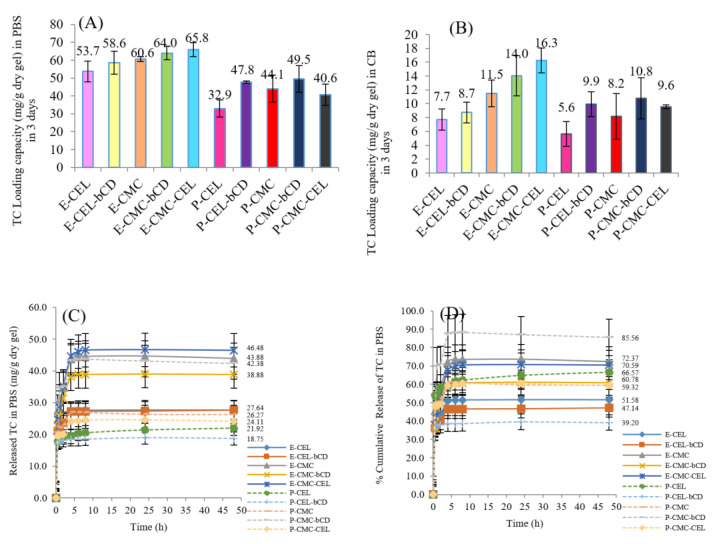

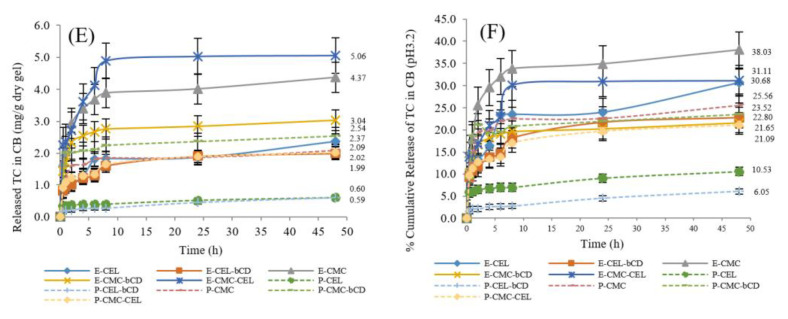
Tetracycline loading amounts in CEL, CEL-βCD, CMC, CMC-βCD and CMC-CEL gels with ECH and PEGDE cross linkers in (**A**) base condition (pH 7.4) and (**B**) acid condition (pH 3.2); Tetracycline release in base condition (pH 7.4) in 2 days: (**C**) cumulative amount of tetracycline release and (**D**) cumulative percentage of Tetracycline release; Tetracycline release in acid condition (pH 3.2) in 2 days: (**E**) cumulative amount of tetracycline release and (**F**) cumulative percentage of Tetracycline release.

**Figure 12:**
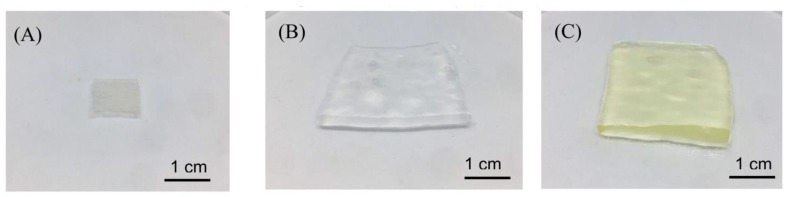
E-CMC-CEL hydrogel (**A**) before swelling, (**B**) swelling in ultrapure water at 25 °C for 2 h, and (**C**) swelling in tetracycline solution at 40 μg mL^−1^ initial TC concentration at 25 °C for 2 h.

TC loading capacities in hydrogels within 3 days at different pH; (1) PBS solution pH 7.4 and (2) citrate buffer pH 3.2 were shown in Figure 11A,B, respectively. From Figure 11A, TC loading of E-CMC-CEL in a basic condition (pH 7.4) had the highest capacity of 65.85 mg g^−1^ dry gel. E-CMC-CEL could store TC more than other types of hydrogels. This was mainly because of the suitable hydroxyl functional group as well as the superior swelling properties of E-CMC-CEL compared with other hydrogels. When considering the effect of βCD in hydrogel such as E-CEL-βCD, E-CMC-βCD, P-CEL-βCD and P-CMC-βCD compared with hydrogel without βCD (i.e., E-CEL, E-CMC, P-CEL and P-CMC, respectively), it was observed that having βCD in a hydrogel could facilitate to increase the efficiency of hydrogel in TC drug loading. The findings were in good accordance with a previous work stating that the structure of βCD had the ability to form complexes with Tetracycline [14] which is hydrophobic compounds by thermodynamics-driven forces such as van der Waals interactions as well as hydrogen bonding [46,47]. In addition, due to the inclusion complexation of βCD molecules with guest hydrophobic TC drugs, it has been reported for an enhancement of drug solubility and stability using βCD complexation [48]. From Figure 11B, tetracycline loading of E-CMC-CEL in acidic condition (pH 3.2) had the highest value of only 16.25 mg g^−1^ dry gel. Hydrogel E-CMC-CEL can uphold greater amount of tetracycline than other types of hydrogel. When compared the TC loading capacity at different pH, Figure 11A,B exhibited that tetracycline loading of the hydrogel in the basic condition (pH 7.4) had better loading capacity than in the acidic condition (pH 3.2). The reason was that drug loading in the acidic condition can lead to less protonation of the carboxyl group (–COO^–^) (typical pKa values in the range of 3–5) and the ether group in the hydrogels that caused less swelling efficiency. With increasing pH of the solution (pH >> pKa), more ionization of the carboxylic acid groups takes place. This results in both electrostatic repulsion between the carboxylate (–COO^–^) groups, as well as the expansion of the space network and thus improves the swelling and drug loading behavior [49]. Figure 12 demonstrated neat E-CMC-CEL before and after water swelling and TC loading at 25 °C for 2 h.

For the study of TC controlled release from hydrogels, all 10 types of hydrogels were investigated for the TC release at different pH at pH 7.4) and at pH 3.2 at 25 °C for 2 days. The amount of TC release over the time was monitored as shown in Figure 11C–F. From Figure 11C,D, TC release of hydrogel in PBS buffer under basic condition (pH 7.4) was found to be efficient during the initial period of 0 to 5 h. After the TC release for 2 days, E-CMC-CEL exhibited the highest amount of TC release at 46.48 mg g^−1^ dry gel corresponding to 70.6% cumulative release, followed by E-CMC which showed TC release of 43.88 mg g^−1^ dry gel (72.4% cumulative release) and P-CMC-βCD with 42.38 mg g^−1^ dry gel (85.56% cumulative release). The results agreed with the swelling ratio of the aforementioned hydrogels in which E-CMC-CEL and E-CMC hydrogels showed superior swelling compared with other hydrogels. In addition, an E-CMC-CEL hydrogel which had greater pore size than other hydrogels could render better diffusion of TC from the hydrogel to the surroundings more effectively. However, the findings showed that although P-CMC-βCD had slightly lower TC release amount of 42.38 mg g^−1^ dry gel, the highest percentage of cumulative TC release of 85.6% was achieved from P-CMC-βCD hydrogel. When comparing loading and release of TC of hydrogel at pH 7.4 with previous work, it was reported that the loading and release of tetracycline were only 8 mg g^−1^ and 5.6 mg g^−1^, respectively, (can release only 76% of the drug storage) from the βCD / CMC gels with ECH as a crosslinker [14]. The reason that βCD could enhance the TC release in basic condition was due to the pKa of βCD, which could show high stability under basic conditions [50]. The hydroxyl groups attached to the rim was disclosed to start to deprotonate at higher pH [51]. Depending on the determination method and the location of the hydroxyl groups, the pKa values of the βCDs has been reported between 12.1 and 13.5 [52]. 

From Figure 11E,F, TC release from hydrogel in citrate buffer in the acidic condition (pH 3.2) was less efficient compared with that at basic condition pH 7.4. Slower TC release rate during 0 to 8 h was found at pH 3.2 relative to pH 7.4. For the study of pKa of tetracycline hydrochloride, the conjugated trione system concerning the amide group was accountable for pKa_1_ = 3.3 [53]. The weakly basic conjugated phenolic enone system was reported to take place at pH = 7.3 which could account for a 25% increase in the value of the tetracycline diffusion coefficients, compared to pH = 3. It has been revealed that amino groups turn out to be ionized only at pH > 8, which is well outside the pH thresholds for the human body [54]. After the TC release for 2 days, as demonstrated in Figure 11E,F, E-CMC-CEL showed highest amount of tetracycline release at 5.06 mg g^−1^ dry gel, followed by E-CMC which was released at 4.37 mg g^−1^ dry gel. The result agreed with the water swelling capacity of these two hydrogels according to their physical and chemical properties for water absorption. However, when considering the tetracycline release percentage of hydrogels, it was found that the highest TC release percentage of E-CMC and E-CMC-CEL were 38.03% and 31.11%, respectively, followed by E-CEL givingTC release percentage at 30.68%. When comparing the pH of both from Figure 11C–F, the results demonstrated that the tetracycline release of hydrogel in basic condition (pH 7.4) was considerably better than in acidic conditions (pH 3.2). Due to the macromolecular architecture flexibilization and restructuration promoted by the increase in the release fluid’s temperature, higher cumulative release ratios are registered for the drug-loaded hydrogels at equilibrium. 

Even though the pH of human skin surface ranges between 5.4 and 5.9, it was found to gradually increase with skin depth, and a pH close to neutral (pH 7.4) was observed after skin destruction in an acute wound [55]. The wound as scar tissue becomes acidic during healing and reestablishing the intact stratum corneum [56]. In contrast, the pH in chronic wounds varies between 5.45 and 8.65, due to the alkaline shift [57] caused by dissolved CO_2_, a reduction in oxygen tension and, possibly, by alkaline anions accumulation from bacterial metabolism. Therefore, pH-responsiveness of TC release at different pHs was suitable for wound healing with antibiotic exerting different release kinetics which will be perfectly matched for each wound type [58]. 

Thermal analysis of material using DSC was able to appraise first and second order thermal transitions, including melting (T_m_), crystallization (T_c_) and glass transition (T_g_) phenomena. It was reported for DSC analysis that pure CMC showed a sharp endothermic peak at 87.14°C and an exothermic peak at 279.86°C [59], while cellulose exhibited T_g_ near the region of − 40 °C, which indicates the water content in the specimen. Another report found an endothermic peak of cellulose at around 80 °C as a consequence of the water evaporation and the inner volatile substances. After cryo-induced E-CMC-CEL hydrogel formation, sharp exothermic peaks were prominent at 178 and 183.8 °C due to crystallization, while a glass transition peak (T_g_) was found at 43.5 °C, as demonstrated in Figure S3. Vanishing or shifting endothermic peaks of reactants in the polymeric network and an appearance of a broad exothermic peak at 178 and 183.8 °C can represent chemical modifications in the polymeric linkage due to higher crosslinking density [60] and branching, leading to complex formation, enhanced stability and higher T_g_ [61,62]. The XRD pattern of an E-CMC-CEL hydrogel (Figure S4) additionally confirmed the complexation of polymer network among carboxymethyl cellulose (CMC), cellulose and epichlorohydrin (ECH) crosslinking agent. Several sharp and clear diffraction signals of dry E-CMC-CEL hydrogel at 2θ = 15, 17.8 and 22.8°, which indicated the XRD diffraction of CMC and cellulose, were disappeared [63]. Instead, the intensity of peak at 28.1°, 32.4°, 46.1°,56.9°, 66.7° and 75.9° was substantially exhibited, which suggested that the chemical crosslinking between the CMC, cellulose and ECH could destroy the crystallization of the cellulose and CMC and in turn generated the crystalline region in the Cellulose/CMC/ECH in E-CMC-CEL hydrogel. The XRD result was in good accordance with previous works [64,65].

### 2.7. Antibiotic Delivery Platform and Antimicrobial Activity of Hydrogels

**Figure 13:**
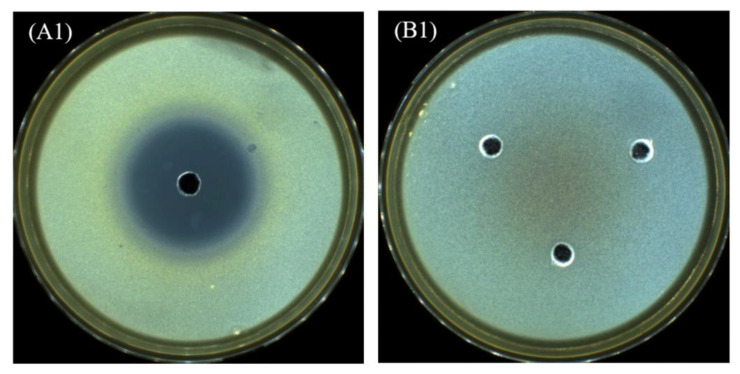

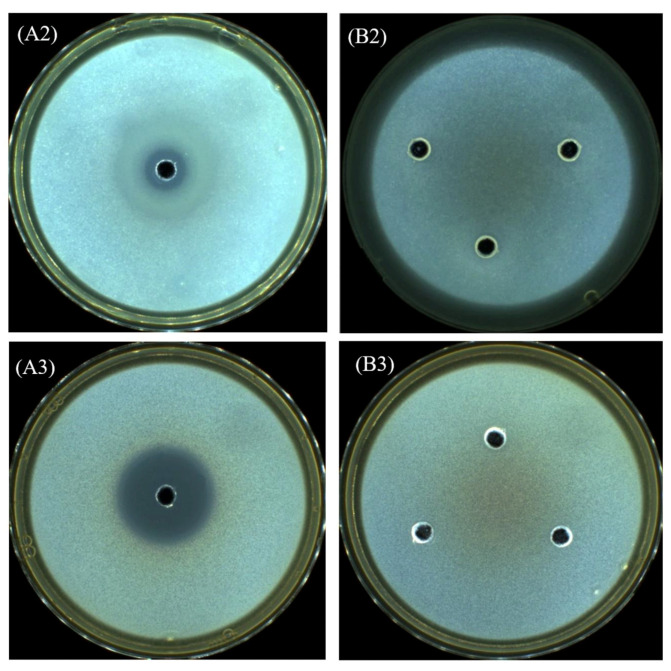
Antimicrobial susceptibility test using disk diffusion method for sample (**A**) E-CMC-CEL hydrogel and (**B**) TC loaded E-CMC-CEL hydrogel at initial 40 μg mL^−1^ tetracycline concentration in PBS buffer pH 7.4 at 25 °C for 2 h toward microbials: (**1**) *Staphylococcus aureus* ATCC 29213, (**2**) *Pseudomonas aeruginosa* ATCC 27853 and (**3**) *Escherichia coli* ATCC 25922.

Neat E-CMC-CEL hydrogel, water swelling E-CMC-CEL hydrogel (negative control) and TC loaded E-CMC-CEL hydrogels as an antibiotic delivery platform were prepared at 25 °C for 2 h for an antimicrobial activity test, as demonstrated in Figure 12. After gel solubilization in medium, the antimicrobial susceptibility test using the disk diffusion method was first examined for a known amount of E-CMC-CEL hydrogel and TC loaded E-CMC-CEL hydrogel toward the growth of *Staphylococcus aureus* ATCC 25923, *Pseudomonas aeruginosa* ATCC 27853 and *Escherichia coli* ATCC 25922, as demonstrated in Figure 13. From the results, tetracycline loaded E-CMC-CEL hydrogel was found to be the strongest at inhibiting the growth of *S. aureus > E. Coli > P. aeruginosa* with a clear zone diameter developed at 30 mm, 19 mm and 10 mm, respectively (Table 1). The greater inhibition zone observed properly describes the antimicrobial potency of tetracycline and, moreover, indicates susceptibility of TC toward microbial cells. To further investigate whether there was synergistic inhibitory effect of the concentration of E-CMC-CEL hydrogel and TC loaded E-CMC-CEL hydrogel, the micro-dilution checkerboard method was used to quantify the minimal inhibitory concentrations, MICs, and the minimum lethal concentration, MLCs, of individual microorganism. The results of MICs and MLCs in which at least eight concentrations of antibiotics representing therapeutically achievable ranges were tested against each organism, as demonstrated in Table 1. It was revealed that TC loaded E-CMC-CEL hydrogel showed significant effect of antimicrobial activity especially toward *S*. *aureus* and *E. coli,* while less inhibitory effect was found toward *P. aeruginosa*. Since the pH of cultivation broth was maintained at pH 7.3 ± 1, there was thus no effect of TC and hydrogel dissociation or deprotonation. Consequently, the difference in inhibitory result was possibly due to antimicrobial resistance to TC of *P. aeruginosa*, and thus higher concentration of TC was required to inhibit the growth of *P. aeruginosa*. No suppression and inhibitory effect of neat E-CMC-CEL hydrogel was observed toward all microorganisms tested. The findings presented that E-CMC-CEL hydrogel was a suitable material for an antibiotic drug-carrying platform, providing successful inhibitory effect on *S. aureus, E. coli* and *P. aeruginosa*, respectively.

## 3. Experiment

### 3.1. Materials

Empty fruit bunch (EFB) of *Elaeis guineensis* or oil palm, the most abundant agricultural residue of palm oil processing [66], was provided from a Palm Oil Mill in Chumphon Province, Thailand. Sodium hydroxide (analytical grade) and urea were obtained from Ajax. Hydrochloric acid (analytical grade) was obtained from RCL Labscan. Monochloroacetic acid (analytical grade) was obtained from Alfa Aesar. Cellulose powder, carboxymethyl cellulose (CMC) powder (CMC, 250,000 g mol^−1^), epichlorohydrin (ECH), polyethylene glycol diglycidyl ether (PEGDE), hydroxyethyl cellulose (HEC), starch and tetracycline (TC) were purchased from Sigma-Aldrich. β-cyclodextrin (βCD) was purchased from Tokyo Chemical Industry. All chemicals were used as received without modification.

### 3.2. Cellulose Production from EFB

EFB was washed, sundried and after that, it was cut, milled and sieved to specific particle size (+50/−200 mesh). The final EFB powder was then dried at 105 °C overnight and kept in an automatic desiccator (25% RH). For cellulose fractionation using hydrothermal reaction, 100 g EFB was mixed with 2 L distilled water in a 15 L pressurized hydrothermal reactor. The hydrothermal reaction was performed at varied temperatures of 180, 200 and 220 °C and 3 MPa for 1 h. After that, the solid fraction was delignified in 2 L of 0.5 M NaOH solution for 4 h at room temperature (30 °C). For further lignin and hemicellulose removal, 2 L of water was added into solid fraction after filtration and the hydrothermal reaction was repeated in an autoclave at 121 °C, 15 psi for 15 min. To obtain high purified cellulose product, repeated bleaching of the residual solid fraction was conducted in 30% *w*/*w* H_2_O_2_ at pH 11.5 with a solid-to-liquid ratio of 1:20 at 60 °C for 1 h. The bleaching solution was changed every bleaching step until the brightness of solid residue was as much as cellulose powder. The brightness of purified cellulose was evaluated by IMAGE J 1.53k software (National Institutes of Health, USA). 

### 3.3. Synthesis of CMC from Cellulose Derived from EFB

To synthesize CMC from purified cellulose, 15 g cellulose was added into a mixture of 50 mL of NaOH and 450 mL of isopropanol. The reaction took place at room temperature (30 °C) for 30 min. Afterward, monochloroacetic acid was added into the mixture and then stirred for 3 h [67]. The effect of NaOH concentration (15–25% *w*/*v*) and monochloroacetic acid (MCA) (2–4% *w*/*v* corresponding to 10–20 g) as well as etherification temperature (50–70 °C) on degree of substitution (DS) of synthesized CMC was investigated. After the reaction, the liquid phase was discarded and the solid phase was suspended in 100 mL of 70% *w*/*w* methanol. The solid phase suspended in methanol was filtered and washed 5 times with 300 mL of 70% *w*/*w* ethanol. It was then washed with methanol and filtered. The residue from the filtration was dried at 55 °C in an oven overnight and the resulting CMC was obtained.

### 3.4. Cryo-Induced Synthesis of Cellulose Based Hydrogel

#### 3.4.1. Influence of Cellulose Derivatives and Crosslinker on Cryogelation

For cellulose-based hydrogel synthesis, cellulose was firstly disintegrated and dissolved in NaOH/urea solution. Cellulose solution (Cell Soln) was prepared by mixing 3 g cellulose in 90 g of solution containing 6 wt% NaOH, 4 wt% urea and 90 wt% distilled water [12]. The mixture was well stirred at 400 rpm for 5 min, and afterward cryogelation was initiated by freezing cellulose solution at −20 °C for 12 h. Then, the frozen mixture was immediately melted to room temperature and subsequently stirred at the rotating speed at 1500 rpm for 10 min. For CMC solution preparation (CMC Soln), CMC was dissolved in NaOH/urea at the same ratio as cellulose solution. Then, cellulose solution (15 g) and CMC solution (15 g) were mixed, and finally epichlorohydrin, a crosslinker, was added. The effect of the amount of epichlorohydrin added at 1, 3 and 5 mL on the chemical and physical properties of hydrogels was investigated. After mixing, the mixture was stirred at 400 rpm at room temperature for 2 h. Synthesized hydrogel was incubated at 60 °C in an oven for 12 h. Influence of different cellulose derivative, i.e., hydroxyethyl cellulose (HEC), and the starch, apart from the CMC solution, was studied.

#### 3.4.2. Effect of Cellulose-to-CMC Ratio and Types of Crosslinking Agents on Hydrogel Properties

To study the effect of cellulose-to-CMC ratio and types of crosslinking agents on hydrogel properties, 3 wt% cellulose solution (Cell Soln) and 3 wt% CMC solution (CMC Soln) were separately prepared in NaOH-Urea solution (90 wt% distilled water/6 wt% NaOH/4 wt% urea). Two solutions (Cell soln + CMC soln) were mixed together at different weight ratios (i.e., 1:9, 2:8, 3:7, 4:6, 5:5 as shown in Table S1) to obtain final weight of mixture at 30 g. After stirring at 400 rpm to gain a homogenous solution, the mixture was frozen at −20 °C for 12 h. After freezing, Cell soln and CMC soln mixture from −20 °C were thawed to room temperature was stirred with 1500 rpm to obtain a transparent solution. After that, the mixture was stirred at 30 °C for 2 h to obtain a homogeneous solution, and then kept in oven at 60 °C for 12 h for complete cryogelation process.

### 3.5. Characterization of Purified Cellulose from EFB and Synthesized CMC

Compositional analysis of cellulose derived from EFB was conducted. Natural detergent fiber (NDF), acid detergent fiber (ADF) and acid detergent lignin (ADL) were quantified and used for calculation of cellulose, hemicellulose and lignin according to Goering method [68]. Cellulose recovery from EFB, cellulose yield and cellulose purity from bleaching were calculated based on dry weight of extracted cellulose compared with raw material (EFB) as shown in Equations (1) to (3).

(1) $$\mathrm{Cellulose}\;\mathrm{recovery}\;\mathrm{from}\;\mathrm{fractionation}\;\left(\%\right)=\frac{Solid\;yield\;\left(\%\right)\times cellulose\;content\;\left(\%\right)}{EFB\;weight\;\left(g\right)\times 100}\;\;\;\;$$

(2) $$\mathrm{Cellulose}\;\mathrm{yield}\;\mathrm{after}\;\mathrm{bleaching}\;\left(\%\right)=\frac{Purified\;cellulose\;\left(g\right)}{EFB\;weight\;\left(g\right)\times Cellulose\;content\;\left(\%\right)/100}\times 100\;$$

(3) $$\mathrm{Cellulose}\;\mathrm{purity}\;\mathrm{after}\;\mathrm{bleaching}\;\left(\%\right)=\frac{cellulose\;\left(wt\%\right)}{bleached\;sample\;\left(g\right)\times 100}$$

In case of CMC analysis, degree of substitution (DS) was measured to indicate the replacement of methoxyl and carboxyl groups in cellulose structure. For DS analysis, 4 g of CMC was added to 75 mL of 95% ethanol and 5 mL of nitric acid (65%). Next, the slurry was boiled for 5 min. The liquid solution was decanted using vacuum pump and washed with 80% ethanol at 60 °C repeatedly until all the acid was removed. Finally, the solid was washed with methanol and filtered by air blown vacuum system until the alcohol was removed. Next, the filter was dried at 105 °C for 3 h and cooled in a desiccator for 30 min. Then, 0.5 g of dry CMC was mixed with 50 mL distilled water and 12.5 mL of 0.3 mol L^−1^ NaOH solution under stirring and heating for complete dissolution. Finally, the mixture was titrated with 0.3 mol L^−1^ HCl to a phenolphthalein end point. The DS of CMC were determined using the following Equation (4) in [69] and Equation (5).

(4) DS =(0.162 × A)/(1 − 0.058 A)

(5) $$A\;=\frac{\mathrm{BC}\;-\mathrm{DE}}{F}$$

where A: milli-equivalents of used HCl per dry weight of specimen (g), the required weight of alkali per gram of sample, B: volume of NaOH solution (mL), C: the normality of NaOH solution (N), D: volume of HCl consumed (mL), E: the normality of HCl solution (N) and F: the dry weight of sample (g). The factor 162 is the molecular weight of the anhydrous glucose unit and 58 is the net increment in the anhydrous glucose unit for every substituted carboxymethyl group. Functional groups of cellulose and CMC synthesized were characterized using Fourier transformed infrared spectroscopy (FTIR). The untreated, pretreated and bleached EFB as well as synthesized CMC were subjected to structural analysis through Spectrum One FTIR spectrophotometer (Nicolet 6700, Thermo scientific, USA). KBr pellet technique was employed in this analysis, wherein the pretreated EFB was ground with analytical grade KBr and pelletized in a hydraulic press. Transmission mode was used for FTIR measurement at the wave numbers ranged between 4000 and 400 cm^−1^ with 100 numbers of scan [16]. 

### 3.6. Characterization of Hydrogels

The functional groups of hydrogels were determined by Fourier transform infrared spectroscopy (FT-IR) (Nicolet 6700, Thermo scientific, USA) in wavenumbers 4000–400 cm^−1^ and 100 numbers of scan. The hydrogel samples were cut into 1.0 cm × 1.0 cm × 0.5 cm in dimension and dried in the vacuum oven at 50 °C at 340 Pa for 24 h. The structure of hydrogels was determined by Scanning electron microscope (SEM Model JEOL JSM-IT500HR, Republic of Korea). Prior to SEM image analysis, dried hydrogel samples were swelling to equilibrium in an excess volume of distilled water at room temperature (25 °C) for 72 h, subsequently frozen in refrigerator at −20 °C for 3 h and then freeze-dried at 50 °C at 133.33 Pa for 24 h. The fracture surface (cross-section) of the hydrogel was coated with gold, and then observed and photographed by SEM at different magnifications. Differential Scanning Calorimeter (DSC 214 Polyma, Netzsch, USA) was analyzed for glass transition, melting point and boiling point temperature of hydrogel under 40 mL min^−1^ nitrogen flow rate atmosphere at 10 °C min^−1^ scan rate from −40 to 260 °C. The X-ray diffraction (XRD) pattern of the substance was measured with Bruker X-ray diffractometer (Model D8 Discover, Bruker, Germany) with 2-theta ranged from 10 to 50 degrees. 

### 3.7. Hydrogel Swelling Measurement

All the synthesized hydrogels were cut into 1.0 cm × 1.0 cm × 0.5 cm in dimension and dried in the vacuum oven at 50 °C at 340 Pa for 24 h. The dried hydrogel samples were swelled in 60 mL distilled water at room temperature (25 °C) for 72 h. Measurement of the change of soaked hydrogel’s weight was performed at 3 min, 8 min, 15 min, 30 min, 45 min, 1 h, 1.5 h, 3 h, 6 h, 9 h, 24 h and 72 h. The percentage of swelling ratio (SR) of hydrogels was calculated based on Equation (6).

(6) $$\%\mathrm{Swelling}\;\mathrm{ratio}\;(\mathrm{SR})=\frac{\mathrm{Weight}\;\mathrm{of}\;\mathrm{wet}\;\mathrm{hydrogel}\;(g)}{\mathrm{Weight}\;\mathrm{of}\;\mathrm{dry}\;\mathrm{hydrogel}\;(g)}\times 100$$

### 3.8. pH Responsive Drug Loading and Release of Hydrogel

All the synthesized hydrogels were cut into 1.0 cm ×1.0 cm ×0.5 cm in dimension and dried in the vacuum oven at 50 °C at 340 Pa for 24 h. The dried hydrogels were swelled in 0.25 mg mL^−1^ tetracycline in different buffers i.e., phosphate buffer solution (pH 7.4) and citrate buffer solution (pH 3.2) at 25 °C for 72 h. The loading capacity of hydrogels was calculated according to Equation (7).

(7) $$\mathrm{Loading}\;\mathrm{capacity}\;(g\;g^{-1})=\frac{(V_{1}\;\times \;c_{1})\;-\;(V\;\times \;C)\;}{\mathrm{Weight}\;\mathrm{of}\;\mathrm{dry}\;\mathrm{hydrogel}}$$

where V_1_ is the volume of initial tetracycline solution (mL), c_1_ is the initial concentration of the tetracycline solution (g mL^−1^), V is the remaining volume of the tetracycline solution (mL) and C is the concentration of remaining tetracycline solution after swelling (g mL^−1^). The concentration of initial and remaining tetracycline solution during the time-course of loading was calculated based on the calibration curve of standard tetracycline solution and UV-Vis absorbance (UV-1800, Thermo Fisher Scientific, Japan) at 271 nm. The drug loading study was performed until the accumulative drug loaded in hydrogels reached the constant value.

For the study on drug release, the tetracycline release profile from hydrogel was evaluated in phosphate and citrate buffer solution at 37 °C for 72 h. The controlled release of tetracycline solution during the study was quantified using UV-Vis spectrophotometry. The cumulative release of tetracycline was calculated based on Equation (8).

Cumulative release (%) = (M_t_/M_0_) × 100 (8)

where M_t_ is the remaining concentration of the tetracycline (g mL^−1^) over the time and M_0_ is the initial concentration of tetracycline (g mL^−1^).

### 3.9. Antibiotic Delivery Platform and Antimicrobial Activity of Hydrogels

TC loaded and neat E-CMC-CEL hydrogels were tested for antimicrobial susceptibility test of different concentrations for *Staphylococcus aureus* (ATCC 29213), *Pseudomonas aeruginosa* (ATCC 27853) and *Escherichia coli* (ATCC 25922) by disk diffusion method expressed as diameter of inhibition zone (mm). Broth dilution method expressed as minimum inhibitory concentration (MIC) and minimal lethal concentration (MLC) was conducted according to Clinical and Laboratory Standards Institute (CLSI) M100 Performance Standards for Antimicrobial Susceptibility Testing (30th Edition, 2020).

## 4. Conclusions

Cryo-induced cellulose-based hydrogels are newly attractive biomaterials for future biomedical applications regarding their tunable mechanical properties, e.g., elasticity, viscous characteristics, sol-gel transition or solid-like characters, as well as controllable physical property, e.g., functionality and porosity. In this research, icy crystals developed during lyophilization of sol-gel formation were found to influence the pore size of hydrogels, which led to an antibiotic drug release rate, in combination with varying environmental pH, that influenced the dissociation of tetracycline drug and hydrogel functional groups. The present study revealed key factors that affected the hydrogel structural properties as well as swelling and release characteristics which were the ratio of cellulose toward carboxymethyl cellulose (CMC) and β-cyclodextrin (β-CD) as well as type of crosslinking agent. The addition of CMC was prone to enhance the viscous property of hydrogel over elastic characteristics, in contrast to the addition of cellulose in which a more solid-like property was obtained. The presence of CMC, moreover, enhanced the wall thickness of the hydrogel pores while a proper amount of β-CD can cause pore diameter reduction and increased hydrogel surface area and porosity. E-CMC-CEL was found the most promising hydrogel, providing the highest TC loading in a basic condition (pH 7.4) at a capacity of 65.85 mg g^−1^ dry gel as well as high TC release at 70.6% at pH 7.4. At the same amount of TC, tetracycline loaded E-CMC-CEL hydrogel was found to have the strongest growth inhibition of *S. aureus > E. coli > P. aeruginosa* according to the clear zone diameter developed. E-CMC-CEL pH-responsive TC release hydrogel at pH 7.4 was apparently matched for chronic wound and bacterial infection wound in which an alkaline shift occurred, and thus an increase in pH in the aforementioned wound type could stimulate a rapid kinetic release rate of antibiotics.

Using an epichlorohydrin crosslinker, which could generate more solidified hydrogel compared with a poly (ethylene glycol) diglycidyl ether (PEGDE) crosslinker, which provided more liquid-like hydrogel, was claimed to be appropriate for use as a filling agent for forthcoming chronic wound-healing application. The findings additionally suggested that vacuum dried cellulose-based hydrogel provided hydrogels suitable for antibiotics and a protein delivery platform, while lyophilized cellulose-based hydrogels, so called cryogels, are proper for assisting cell growth and cell proliferation due to their superior porosity and more rigid structure, which are promising aspects for further study.

## Figures and Tables

**Table 1:** Antimicrobial activity test of tetracycline loaded E-CMC-CEL and swelled E-CMC-CEL hydrogels.

Samples	Microorganisms	Antimicrobial Test		
		Diameter of Inhibition Zone (mm)	MICs(%)	MLCs(%)
E-CMC-CEL hydrogel ^a^	*Staphylococcus aureus* ATCC 29213	0.0	>100.00	>100.00
	*Pseudomonas aeruginosa* ATCC 27853	0.0	>100.00	>100.00
	*Escherichia coli* ATCC 25922	0.0	>100.00	>100.00
TC loaded E-CMC-CEL hydrogel ^b^	*Staphylococcus aureus* ATCC 29213	30.0	0.024	6.250
	*Pseudomonas aeruginosa* ATCC 27853	10.0	12.500	50.000
	*Escherichia coli* ATCC 25922	19.0	0.195	12.500

^a^ E-CMC-CEL was prepared from CMC soln:Cell Soln = 9:1 with ECH crosslinking agent and swelling in ultrapure water at 25 °C for 5 h. ^b^ TC loaded E-CMC-CEL hydrogel at initial 40 μg mL^−1^ tetracycline in PBS buffer pH 7.4 at 25 °C for 5 h.

## Data Availability

The data presented in this study are available on request from the corresponding author.

## Supplementary Materials

The following supporting information can be downloaded at: https://www.mdpi.com/article/10.3390/ijms24021230/s1.
