# Supplementary material for: Cryo-Induced Cellulose-Based Nanogel from Elaeis guineensis for Antibiotic Delivery Platform
Source: Int J Mol Sci. 2023 Jan 8;24(2):1230. doi: 10.3390/ijms24021230 (PMC9866051; doi:10.3390/ijms24021230)
Supplement: Supplementary file 1 [file ijms-24-01230-s001.zip › ijms-2115767-supplementary.pdf]

Cryo-Induced Cellulose-Based Nanogel from *Elaeis guineensis* for Antibiotic Delivery Platform

Tasnim Hajidariyor [1], Nutchanon Nuntawad [1], Panadda Somsaen [1], Raninnart Prukdamrongchai [1], Harit Cherdchoo [1], Pattaraporn Posoknistakul [1], Pongtanawat Khemthong [2], Wanwitoo Wanmolee [2], Pariyapat Arjfuk [3], Pisut Pongchaikul [3], Navadol Laosiripojana [4], Kevin C.-W. Wu [5,6,7,8,9] and Chularat Sakdaronnarong [1,*]

[1] Department of Chemical Engineering, Faculty of Engineering, Mahidol University, Nakorn Pathom 73170, Thailand

[2] National Nanotechnology Center (NANOTEC), National Science and Technology Development Agency (NSTDA), Pathum Thani 12120, Thailand

[3] Chakri Naruebodindra Medical Institute, Faculty of Medicine Ramathibodi Hospital, Mahidol University, Samut Prakarn 10540, Thailand

[4] The Joint Graduate School of Energy and Environment, King Mongkut's University of Technology Thonburi, Bangkok 10140, Thailand

[5] Department of Chemical Engineering, National Taiwan University, Taipei 10617, Taiwan

[6] Center of Atomic Initiative for New Materials (AI-MAT), National Taiwan University, Taipei 10617, Taiwan

[7] International Graduate Program of Molecular Science and Technology, National Taiwan University (NTU-MST), Taipei 10617, Taiwan

[8] Department of Chemical Engineering and Materials Science, Yuan Ze University, Taoyuan 320315, Taiwan

[9] Yonsei Frontier Lab, Yonsei University, Seoul 03722, Republic of Korea

* Correspondence: chularat.sak@mahidol.ac.th

Table S1. Chemical composition of the matrices and crosslinkers for cellulose/CMC hydrogels synthesis

Hydrogels	Matrices		Crosslinkers		
	CMC Sol^n (g)	CEL Sol^n (g)	βCD (g)	ECH (mL)	PEGDE (mL)
E-CMC-CEL55	15	15	-	3	-
E-CMC-CEL64	18	12	-	3	-
E-CMC-CEL73	21	9	-	3	-
E-CMC-CEL82	24	6	-	3	-
E-CMC-CEL91	27	3	-	3	-
E-CMCCEL	27	3	-	3	-
E-CEL	-	30	-	3	-
E-CELβCD	-	27	3	3	-
E-CMC	30	-	-	3	-
E-CMCβCD	27	-	3	3	-
P-CMCCEL	27	3	-	-	3
P-CEL	-	30	-	-	3
P-CELβCD	-	27	3	-	3
P-CMC	30	-	-	-	3
P-CMCβCD	27	-	3	-	3

Table S2. Cellulose recovery from hydrothermal fractionation, cellulose yield and cellulose purity after bleaching

Hydrothermal fractionation	Chemical composition (wt%)					After hydrothermal fractionation				After bleaching (10 times)	
	Cellulose	Hemicellulose	Lignin	Ash	Other	Solid yield (%)	Hemicellulose removal (%)	Lignin removal (%)	Cellulose recovery (%)	Cellulose yield (%)	Cellulose purity (%)
Raw EFB	39.50	23.02	11.71	1.10	24.67	100	na	na	na	na	na
180 °C, 3 MPa, 1h	58.70	2.17	17.63	13.51	7.7	52.27	95.07	21.30	77.68	72.83	99.24
200 °C, 3 MPa, 1h	57.68	2.18	22.53	11.60	6.02	39.12	96.30	24.73	57.13	51.92	92.88
220 °C, 3 MPa, 1h	51.20	3.51	31.17	8.83	5.29	28.91	95.59	23.05	37.47	30.11	85.65

Note: na is not applicable.

Table S3. Whiteness of cellulose characterized by IMAGE J

Condition	Number of bleaching	L*	a	b	ΔE
180 °C, 3 MPa, 1h	Unbleached	3.7	1.0	1.3	4.0
	1	3.9	1.2	1.6	4.4
	2	5.3	3.3	2.2	6.6
	3	7.3	4.0	3.0	8.8
	4	7.4	4.4	3.3	9.2
	5	29.7	14.3	25.3	41.6
	6	30.5	15.2	26.1	42.9
	7	52.0	-2.0	9.3	52.9
	8	54.2	6.6	8.4	55.2
	9	57.6	6.9	2.5	58.1
	10	59.0	7.3	-16.0	61.6
200 °C, 3 MPa, 1h	Unbleached	4.3	1.3	-0.3	4.5
	1	12.3	2.5	2.0	12.7
	2	14.6	5.1	8.9	17.8
	3	15.7	6.3	11.0	20.2
	4	17.1	7.0	11.6	21.8
	5	17.7	8.0	12.3	23.0
	6	18.9	9.0	15.6	26.1
	7	27.0	5.3	17.0	32.3
	8	30.5	5.1	16.0	34.8
	9	31.8	5.6	17.1	36.5
	10	34.0	6.0	19.7	39.8
220 °C, 3 MPa, 1h	Unbleached	3.7	0.3	1.0	3.8
	1	4.1	1.2	1.9	4.7
	2	6.2	2.2	0.6	6.6
	3	7.0	2.3	-1.7	7.6
	4	8.1	3.3	3.4	9.4

	5	8.7	5	6.7	12.1
	6	15.2	7.1	7.9	18.5
	7	19.7	9	9.7	23.7
	8	20.3	9.5	10.5	24.8
	9	20.9	10.4	11.3	25.9
	10	21.3	11.3	12	26.9
Standard cellulose	-	51.3	5.3	4.3	51.8

Table S4. Degree of substitution and whiteness of synthesized CMC from EFB derived cellulose at different CMC synthesis conditions

CMC code	Parameters			Responses				
	NaOH (%w/v)	Monochloroacetic acid (%w/v)	Etherification Temperature (°C)	Degree of substitution (DS)	L*	a	b	ΔE
CMC 1	15	2	50	0.71 ± 0.02	78.9	3.6	5.4	79.2
CMC 2	15	4	50	0.56 ± 0.02	82.3	11.1	5.2	83.2
CMC 3	15	2	70	0.75 ± 0.01	79.7	10.9	7.1	80.8
CMC 4	15	4	70	0.43 ± 0.03	88.8	9.5	6.8	89.6
CMC 5	25	2	50	0.61 ± 0.01	77.3	12.6	7.6	78.7
CMC 6	25	4	50	0.45 ± 0.02	84.0	17.8	8.6	86.3
CMC 7	25	2	70	0.61 ± 0.02	74.6	15.4	6.2	76.4
CMC 8	25	4	70	0.65 ± 0.01	78.1	15.8	9.5	80.2
CMC 9	20	3	60	0.59 ± 0.02	80.7	14.4	9.1	82.5
CMC 10	20	3	60	0.60 ± 0.01	79.2	15.0	8.2	81.0
CMC 11	20	3	60	0.59 ± 0.02	82.1	13.9	8.6	83.7

Table S5. ANOVA of Quadratic Regression of Degree of Substitution (DS) from CMC synthesis

Regression Statistics	
Multiple R	0.899832
R Square	0.809697
Adjusted R Square	0.365657
Standard Error	0.075673
Observations	11

ANOVA

	df	*SS*	*MS*	*F*	*Significance F*
Regression	7	0.073094	0.010442	1.823477	0.335047
Residual	3	0.017179	0.005726		
Total	10	0.090273			

	Coefficients	*Standard Error*	*t Stat*	*P-value*	*Lower 95%*	*Upper 95%*	*Lower 95.0%*	*Upper 95.0%*
Intercept (β_0)	4.497708	2.628125	1.711376	0.185535	-3.86616	12.86157	-3.86616	12.86157
β_3	-0.12675	0.088148	-1.43793	0.246023	-0.40727	0.153775	-0.40727	0.153775
β_{12}	0.00875	0.005351	1.635244	0.200513	-0.00828	0.025779	-0.00828	0.025779
β_{13}	0.000725	0.000535	1.354917	0.268444	-0.00098	0.002428	-0.00098	0.002428
β_{23}	0.000375	0.002675	0.140164	0.897412	-0.00814	0.008889	-0.00814	0.008889
β_{11}	-0.00183	0.000907	-2.01149	0.137784	-0.00471	0.001062	-0.00471	0.001062
β_{22}	-0.04521	0.032463	-1.39263	0.257997	-0.14852	0.058102	-0.14852	0.058102
β_{33}	0.000938	0.000675	1.389722	0.258787	-0.00121	0.003084	-0.00121	0.003084

Note: Regression model is $y = \beta_0 + \beta_3 x_3 + \beta_{12} x_1x_2 + \beta_{13} x_1x_3 + \beta_{23} x_2x_3 + \beta_{11} x_1^2 + \beta_{22} x_2^2 + \beta_{33} x_3^2$, when y is degree of substitution (DS) of synthesized CMC, x_1 is NaOH concentration (wt%), x_2 is monochloroacetic acid (MCA) (%w/v), and x_3 is etherification temperature (°C).

Table S6. ANOVA of Quadratic Regression of Whiteness (ΔE) from CMC synthesis

Regression Statistics	
Multiple R	0.951587
R Square	0.905517
Adjusted R Square	0.685057
Standard Error	2.074046
Observations	11

ANOVA

	df	*SS*	*MS*	*F*	*Significance F*
Regression	7	123.6805	17.66864	4.107393	0.136529
Residual	3	12.905	4.301667		
Total	10	136.5855			

	Coefficients	*Standard Error*	*t Stat*	*P-value*	*Lower 95%*	*Upper 95%*	*Lower 95.0%*	*Upper 95.0%*
Intercept (β_0)	-33.375	72.03174	-0.46334	0.674668	-262.612	195.8621	-262.612	195.8621
β_3	3.80625	2.415952	1.575466	0.21323	-3.88239	11.49489	-3.88239	11.49489
β_{12}	-0.035	0.146657	-0.23865	0.826749	-0.50173	0.431729	-0.50173	0.431729
β_{13}	-0.041	0.014666	-2.79564	0.068099	-0.08767	0.005673	-0.08767	0.005673
β_{23}	0.0125	0.073329	0.170466	0.875492	-0.22086	0.245864	-0.22086	0.245864
β_{11}	0.057125	0.024867	2.297227	0.105256	-0.02201	0.136263	-0.02201	0.136263
β_{22}	0.495833	0.889734	0.557283	0.616213	-2.3357	3.327363	-2.3357	3.327363
β_{33}	-0.02524	0.018489	-1.36509	0.265583	-0.08408	0.033602	-0.08408	0.033602

Note: Regression model is $y = \beta_0 + \beta_3 x_3 + \beta_{12} x_1x_2 + \beta_{13} x_1x_3 + \beta_{23} x_2x_3 + \beta_{11} x_1^2 + \beta_{22} x_2^2 + \beta_{33} x_3^2$, when y is whiteness (ΔE) of synthesized CMC, x_1 is NaOH concentration (wt%), x_2 is monochloroacetic acid (MCA) (%w/v), and x_3 is etherification temperature (°C).

Table S7. Reaction conditions and chemical composition of cryo-induced cellulose-based hydrogels

Sample code	Cell Soln : CMC Soln 15 g : 15 g	Epicholohydrin (mL)	Successful Cryogelation
CMC-E1	Cellulose + CMC	1	×
CMC-E3	Cellulose + CMC	3	✓
CMC-E5	Cellulose + CMC	5	✓
HEC-E1	Cellulose + HEC	1	×
HEC-E3	Cellulose + HEC	3	✓
HEC-E5	Cellulose + HEC	5	×
STA-E1	Cellulose + Starch	1	×
STA-E3	Cellulose + Starch	3	×
STA-E5	Cellulose + Starch	5	×

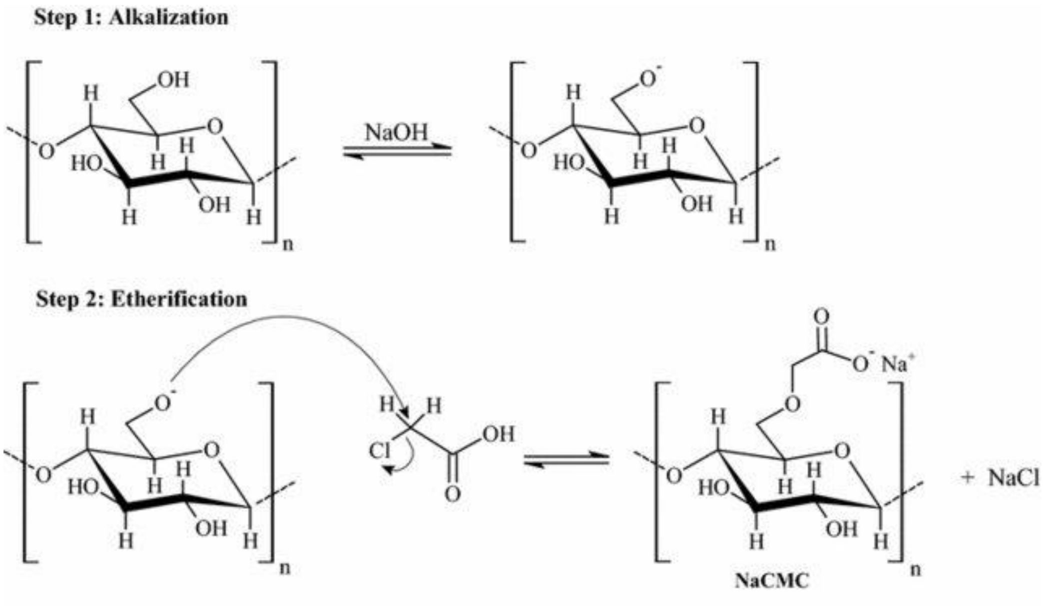

Scheme S1. CMC synthesis from cellulose: Step 1 Alkalization or Mercerization and Step 2 Etherification.

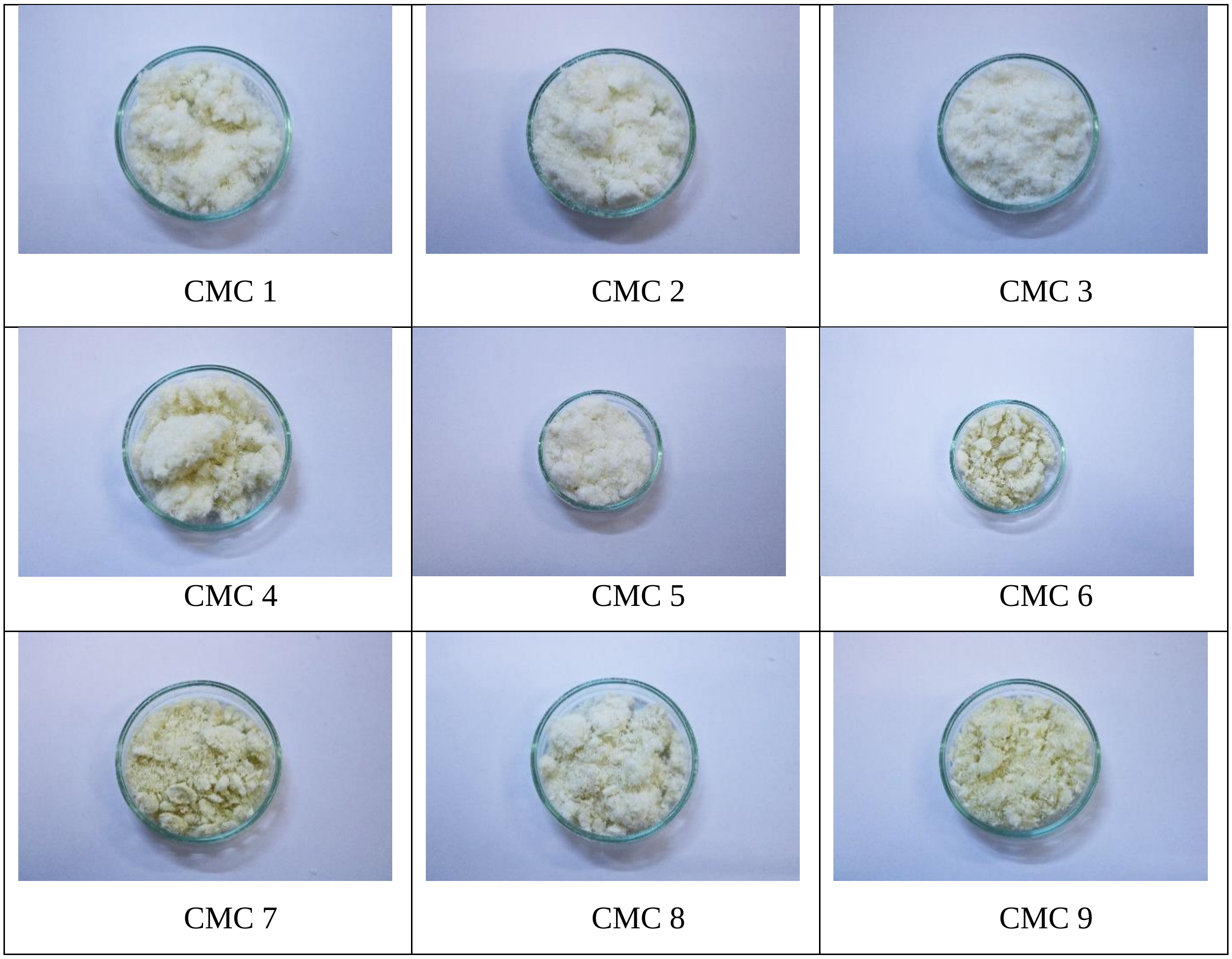

Figure S1. CMC from EFB derived cellulose from different synthesis conditions.

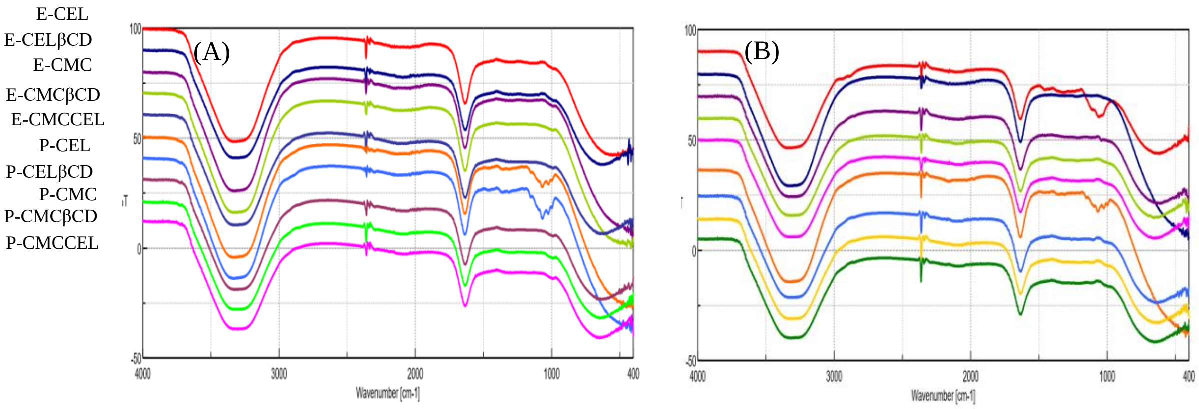

Figure S2. FT-IR spectra of the hydrogels using ECH and PEGDE as a cross-linker after loading of in 0.25 mg mL^{-1} Tetracycline A) at pH 7.4, and B) at pH 3.2 at 25 °C for 3 days.

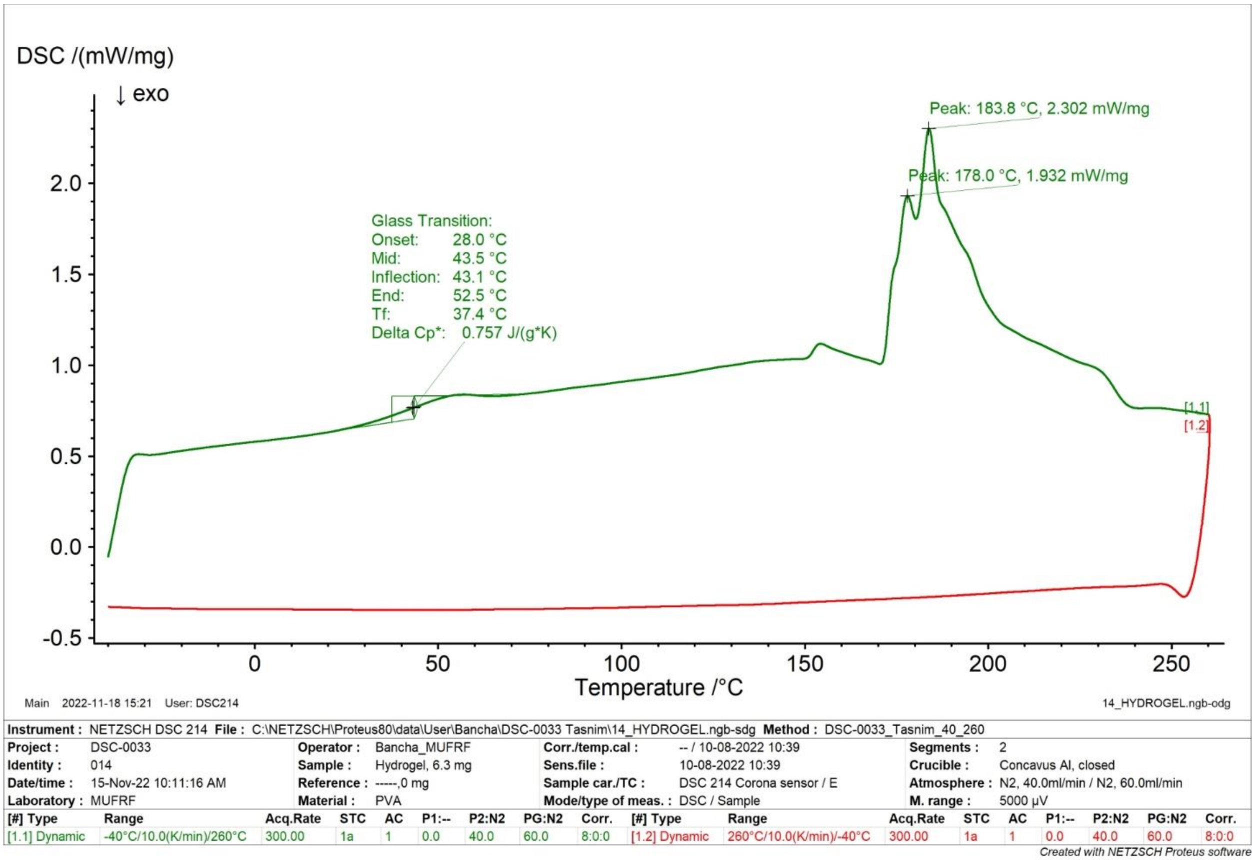

Figure S3. Differential Scanning Calorimetry (DSC) of E-CMC-CEL hydrogel.

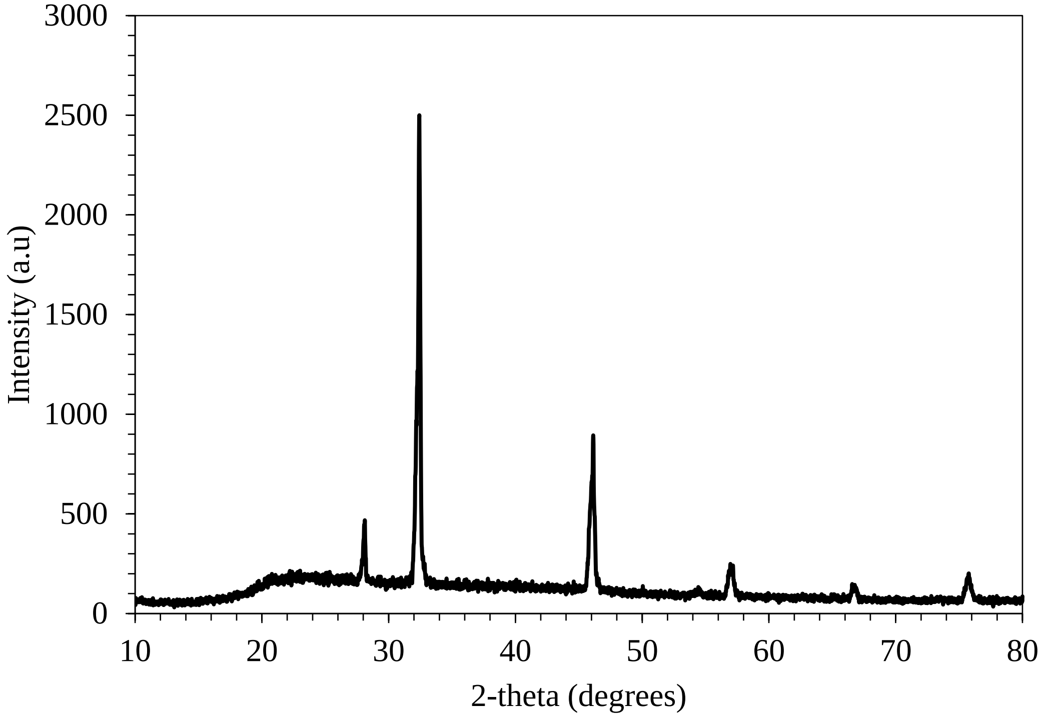

Figure S4. XRD diffraction pattern of E-CMC-CEL hydrogel after freeze drying.
